# Intensified return to work clinical aftercare for sick-listed employees with diagnosed mental disorders in Germany: effects of a multicentre randomized controlled trial

**DOI:** 10.1186/s12888-026-08639-0

**Published:** 2026-09-24

**Authors:** Fiona Starke, Johannes Zimmermann, Alexandra Sikora, Inge Houkes, Anika Schulz, Ralf Stegmann, Laura Seufert, Eva Schmitz, Claudia Buntrock, Gregor R. Szycik, Anna Borgolte, Hans-Peter Unger, Jan Ole Schumacher, Heiko Stark, Carlotta Schneller, Sarah-Louise Unterschemmann, Angelique de Rijk, Uta Wegewitz

**Affiliations:** 1 Division 3 Work and Health, Unit 3.5 Evidence-based Occupational Health, Workplace Health Management, Federal Institute for Occupational Safety and Health, Nöldnerstr. 40-42, 10317 Berlin, Germany; 2 Department of Social Medicine, Faculty of Health, Medicine and Life Sciences, CAPHRI Care and Public Health Research Institute, Maastricht University, P.O. Box 616, Maastricht, MD, 6200 Netherlands; 3 Department of Psychology, University of Kassel, Holländische Str. 36-38, 34127 Kassel, Germany; 4 Institute of Social Medicine and Health Systems Research, Medical Faculty, Otto-von-Guericke University Magdeburg (OVGU), Leipziger Str. 44, 39120 Magdeburg, Germany; 5 Department of Psychiatry, Social Psychiatry and Psychotherapy, Hannover Medical School, Podbielskistr. 162, 30625 Hanover, Germany; 6 Department of Psychiatry, Psychotherapy and Psychosomatics, Centre for Mental Health, Asklepios Clinic Harburg, Eißendorfer Pferdeweg 52, 21075 Hamburg, Germany; 7 Department of Psychiatry, Burghof-Clinic, Ritterstr. 19, 31737 Rinteln, Germany; 8 Department of Psychiatry, Psychotherapy and Psychosomatics, Alexian St. Joseph-Hospital Berlin-Weissensee, Gartenstr. 1, 13088 Berlin, Germany; 9 Department of Psychiatry, Clinic Wittgenstein, Sählingsstr. 60, 57319 Bad Berleburg, Germany; 10 Department of Psychology, Philipps University of Marburg, Gutenbergstraße 18, 35037 Marburg, Germany; 11 Division 3 Work and Health, Federal Institute for Occupational Safety and Health, Nöldnerstraße 40-42, 10317 Berlin, Germany

**Keywords:** Mental disorders, Aftercare, RCT, Employees, Return to Work, Intervention, Clinical treatment, Sickness absence

## Abstract

**Background:**

Mental disorders are a leading cause of sickness absence and disability in Europe. They often follow chronic or recurrent patterns, hindering a sustainable return to work (RTW). In Germany, RTW aftercare interventions focusing on maintaining recovery and minimizing relapse among employees transitioning from psychiatric treatment to the workplace are scarce. We therefore developed an intensified RTW clinical aftercare program (RTW-PIA). This study aimed to (*i*) evaluate the effectiveness of RTW-PIA in addition to Care as Usual (CAU) on achieving sustainable RTW (SRTW) compared to CAU only, (*ii*) assess its impact on secondary outcomes, and (*iii*) explore potential moderators.

**Methods:**

This multicentre randomized controlled trial, conducted in psychiatric outpatient clinics, included sick-listed employees diagnosed with a mental disorder. Participants (*N* = 484) were randomized to RTW-PIA + CAU (*n* = 240) or CAU only (*n* = 244). RTW-PIA is an 18-month multimodal aftercare combining medical-psychotherapeutic and work-oriented components. Primary outcome was self-reported SRTW, defined as ≤ 6 weeks of sickness absence within 12 months after full RTW. Primary and secondary outcomes (e.g., RTW self-efficacy, work ability) were collected at baseline and at 6, 12, 18, and 24 months. Intention-to-treat analyses used logistic regression and growth curve models, complemented by prespecified sensitivity and exploratory moderator analyses.

**Results:**

Overall, RTW-PIA had no significant effect on achieving SRTW (OR = 1.38 [95% CI 0.89 to 2.12]). Secondary outcomes revealed no significant effects, despite a consistent pattern of slight improvements favoring RTW-PIA. Per-protocol and complete-case sensitivity analyses led to the same conclusion. In diagnostic subgroup sensitivity analyses, employees with recurrent depressive disorders showed significant improvements in several secondary outcomes. Prespecified exploratory moderator analyses suggested potential benefits for employees without a recognized disability degree and for those with low social support at work. After multiplicity adjustment, only recurrent depressive disorder still moderated the treatment effect on work ability.

**Conclusions:**

On average, RTW-PIA did not significantly improve SRTW or secondary outcomes. Employees without a recognized disability degree, with low social support at work, or with diagnosed recurrent depressive disorders might benefit from RTW-PIA. These hypotheses need to be tested in independent samples. Further research should also focus on the implementation of RTW-PIA.

**Trial registration:**

DRKS00026232 (prospectively registered on 01/09/2021).

**Supplementary Information:**

The online version contains supplementary material available at https://doi.org/10.1186/s12888-026-08639-0.

## Background

Mental disorders are widespread globally and represent a major driver of sickness absence and disability in Europe [1]. Given their often chronic, relapsing, or recurrent nature, they frequently result in prolonged illness episodes, repeated sickness absence, and multiple attempts to return to work (RTW), all of which impair occupational functioning [2–4]. Thus, employees with mental disorders are more likely to face job insecurity, unemployment, or even permanent exit from the workforce [5–7]. In Germany, where this study was conducted, mental disorders had the highest average duration of sickness absence in 2024 (32.9 calendar days [8]) and accounted for about 42% of all disability benefits in 2023 [9]. Because mental disorders place a substantial burden on individuals and their families, as well as on healthcare systems, labor markets, and society as a whole [10], greater emphasis on and investment in aftercare treatment programs and sustainable RTW (SRTW) practices are more important than ever.

For employees who have been diagnosed with and treated for a mental disorder, RTW occurs at the intersection of the mental health care system and the workplace. This makes their RTW process complex [11]. RTW efforts for this group should go beyond symptom alleviation in treatment, as increased vulnerability and functional limitations may persist despite clinical remission [12]. Therefore, SRTW interventions following discharge from psychiatric inpatient or day clinics ought to be developed as a continuous, integrated treatment and support process rather than a one-time therapeutic strategy [13]. Integrating structured aftercare into hospital discharge management could be crucial for maintaining recovery and supporting SRTW, thereby minimizing relapses [12, 14].

According to a systematic review of successful RTW determinants, a combination of medical-psychotherapeutic and work-oriented measures (e.g., work modification, problem solving) appears to be particularly promising [15]. Contact with the workplace, gradual RTW, and the utilization of multicomponent interventions were found to be essential components for success [16]. To date, clinical and work-directed interventions aiming at SRTW are scarce, and their implementation and effectiveness remain underexplored.

In Germany, both RTW support and sustainable work participation have not yet been routinely included in psychiatric aftercare treatments. For this reason, we developed and evaluated an 18-month intensified RTW aftercare intervention, referred to as RTW-PIA. RTW-PIA comprises three fundamental modules - face-to-face individual RTW support, monthly RTW aftercare group meetings, and web-based aftercare - that build on one another throughout the therapeutic treatment and RTW process [17]. These modules are delivered by healthcare providers who play a vital role in facilitating SRTW for individuals with mental disorders [18].

RTW-PIA is based on the Conservation of Resources theory [19], which emphasizes the need for resources in work life to accomplish future tasks and goals using several therapeutic intervention methods: cognitive-behavioral therapy, mentalization-based techniques, stress management, resilience training, mindfulness, systemic therapy, and psychoeducation. These methods were supplemented with work-directed elements, e.g., the ‘four-phase model of vocational reintegration’ that helps clinicians with managing the patient´s RTW process [13]. RTW-PIA equips treatment providers with strategies to strengthen the work- and nonwork-related resources of returnees at different levels they are involved with: the individual (I), group (G), leadership (L), organization (O), and overarching context (O). The IGLOO model suggests that higher resource levels promote SRTW, while insufficient resources can lead to absence recurrence [20].

### Objectives

The aims of the study were to (*i*) evaluate the effectiveness of RTW-PIA added to Care as Usual (CAU) on achieving SRTW in employees diagnosed with a mental disorder, compared to CAU only; (*ii*) assess the impact of RTW-PIA on changes in participants’ work ability, RTW self-efficacy, health-related quality of life, job satisfaction, impairments in mental functioning, and psychological symptom severity over a 24-month period; and (*iii*) explore potential moderators to determine which baseline variables may modify the effectiveness of RTW-PIA. We hypothesized that there would be more favorable outcomes for employees undergoing both RTW-PIA and CAU than those receiving CAU only. To avoid repetition, the RTW-PIA intervention delivered alongside CAU will hereafter be abbreviated as “RTW-PIA”.

## Methods

### Context of the study: Germany

#### (Mental) Health Care System

The present study was conducted in Germany, a federal republic comprising 16 federal states. The nation’s social insurance system aims to protect income, and health insurance is mandatory [21]. Mental health care in Germany is organized in inpatient (hospital-based), outpatient, and in rehabilitation sectors [22]. In 2022, the nation´s 131 psychiatric hospital beds per 100,000 inhabitants were nearly twice the OECD average [23]. Psychiatric residential treatment for mental disorders is mainly provided by office-based psychiatrists and psychotherapists. Further details on the German healthcare system are provided in [24].

Hospital-affiliated psychiatric outpatient clinics, where RTW-PIA was executed and assessed, deliver intensive, multidisciplinary care for chronic, severe or complex mental disorders. They focus on individuals who do not qualify for traditional outpatient care (i.e., those with severe conditions or common mental disorders with complicated courses or multiple comorbidities), thereby bridging the gap between inpatient and standard outpatient treatment or serving as an alternative to hospitalization [25, 26].

#### Sickness absence and disability policy

One specific work accommodation to help employees return to work after long-term sickness absence is gradual RTW. This measure is an integrative process in which sick-listed employees voluntarily and gradually resume work under medical oversight for a predetermined duration, while receiving 70% of their salary through social insurance. Gradual RTW often involves multiple stakeholders, such as treating physicians, occupational therapists, health insurance providers, and employers [27]. Some of the mental disorders in our study (e.g., depressive disorders) qualify as disabilities when they result in long-term impairments of participation in society and allow eligibility for additional support (e.g., preventive health measures, therapy, rehabilitation, workplace adaptations) [21, 28, 29]. Further information on sickness absence, disability legislation, and the German labor market context can be found in [24].

**Table 1:** Inclusion and exclusion criteria

Inclusion criteria	Exclusion criteria
• Aged between 18 and 60 years • Diagnosis of a mental disorder within the ICD-10 code range F20–F60 • Being sick-listed ≥ six weeks • Employed for ≥ 15 h per week in the German primary labor market ^1^ • Informed consent of participation in the study	• Diagnosis of organic, including somatic, mental [ICD-10 code F0] or substance abuse disorders [ICD-10 code F1] • Acute, severe somatic disease • Pregnancy • Decision against RTW • Planned and/or approved rehabilitation stay ^2^

^1^ The latter refers to those employed at a public or private employer (thus salaried workers) without public subsidies supporting employment. Individuals were considered employed if they were working either part-time or full-time with a legal employment contract in the primary labor market

^2^ A common publicly insured recovery pathway in Germany

### Study design and participants

This study was conducted between January 2021 and June 2025 in a two-arm, multicentre, randomized, controlled design. It received approval from the principal Ethics Committee at Hannover Medical School (ID: 9824_BO_S_2021) and was prospectively registered (DRKS00026232). CAU and RTW-PIA took place in five participating psychiatric outpatient clinics, embedded in psychiatric departments at general hospitals or specialized hospitals located in rural, urban, and metropolitan areas across four federal states, and selected ahead of the trial using convenience sampling. Multidisciplinary RTW treatment teams (e.g., psychotherapists, psychiatrists, occupational therapists, social workers) were trained in participant recruitment and delivery of RTW-PIA.

**Fig. 1:**
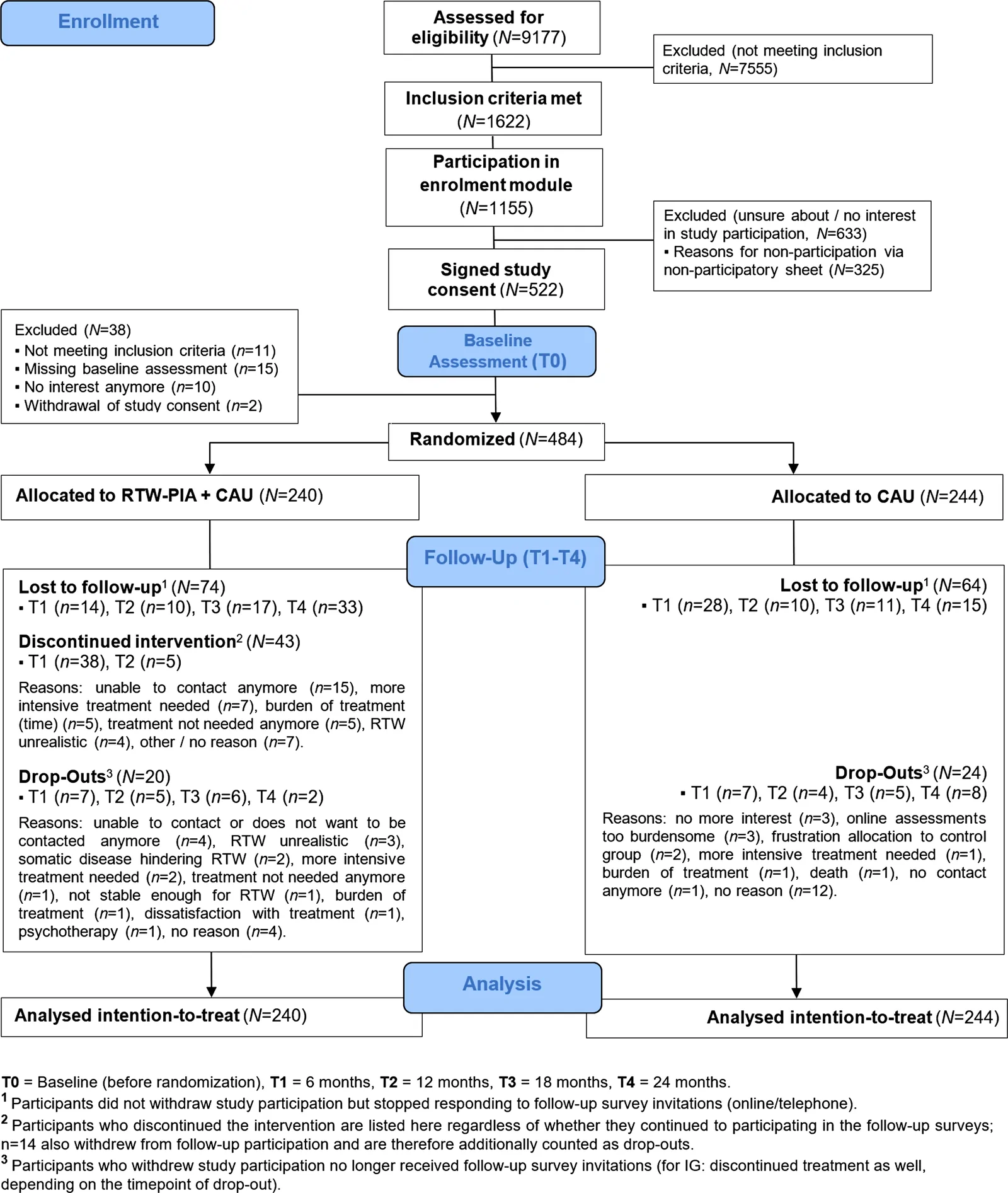
Consort flow chart

Between September 2021 and March 2023, patients were screened across departments at the five participating study centers (inpatient, day-care, and outpatient settings) based on the inclusion and exclusion criteria (Table 1). Eligible patients attended one psychoeducational session titled “Work and Health” with general information about RTW and the interdependency of health and work. Patients were informed about the study and asked about their willingness to participate. All participating patients provided written informed consent. The study protocol can be found elsewhere [17], and reporting follows the CONSORT 2010 guidelines for RCTs [30]. The flow of participants through the study, including enrollment, randomization, allocation, dropout, and analysis, is shown Fig. 1.

### Interventions

#### Care as Usual

CAU was tailored for each participant based on their specific diagnosis, illness severity, and associated impairments. Participants had medical appointments every 4 to 6 weeks and received social worker and psychological counseling to coordinate non-medical treatments like occupational therapy and group therapy. Typically, there were three to five professional contacts each quarter (1–3 medical contacts, 2–4 other contacts). CAU lasted 1–3 quarters on average, depending on clinical severity, and did not specifically focus on work-related issues.

#### RTW-PIA added to Care as Usual

RTW-PIA was an 18-month intervention combining medical and psychotherapeutic approaches with work-directed components. The intervention consisted of three core modules: Individual RTW support (Module A), RTW aftercare group meetings (Module B), and a web-based aftercare (Module C). Modules A and C followed a needs-oriented approach, while Module B was implemented according to a standardized manual. In our study protocol [17], a minimum RTW-PIA dose was defined a priori for all three modules: ≥3 of 8 Module A sessions, ≥ 6 of 9 Module B sessions, and ≥ 1 of 3 Module C video conference sessions. This minimum dose was not empirically derived but was instead pragmatically set to ensure exposure to the core components of this sequential intervention while accommodating varying support needs.

Hence, participants could receive up to *N* = 20 RTW-PIA sessions in total alongside their CAU treatment. Their treatment providers ensured continuity of care throughout. In each study center, CAU was provided by separate teams that did not carry out RTW-PIA. A comprehensive description of the intervention is provided elsewhere [17]; supplementary information can be obtained from the corresponding author.

### Sample size

The study protocol specified a target sample size of *N* = 506 participants, accounting for an anticipated dropout rate of 40% [31], to achieve a power β = 80% for detecting an effect-size of Odds Ratio (OR) = 2 (assuming a 60% probability regarding SRTW achievement in the CAU group). Due to recruitment delays during the COVID-19 pandemic, the target sample size was revised to *N* = 445 participants during recruitment and prior to data analysis. Assuming a 40% dropout rate, a sample size of *N* = 267 participants would provide 80% power to detect an effect size of OR = 2.11, or 74.7% power to detect the originally targeted effect size of OR = 2.

### Outcome measures

Participant data was collected in a pseudonymized form via online questionnaires at five timepoints over 24 months (T0 = baseline, T1 = 6 months, T2 = 12 months, T3 = 18 months, T4 = 24 months).

#### Primary outcome

The primary outcome was the employees’ self-reported achievement of SRTW. SRTW was confirmed by reporting ≤ 6 weeks of contractually agreed working days missed due to sickness absence within 12 months after full RTW. The day after completion of all gradual RTW measures, e.g., the uptake of contractually agreed working days, represented the day of full RTW. The achievement of SRTW was assessed via a self-developed one-item question at 12 (T2), 18 (T3) or 24 months (T4) after baseline and was supported by a variety of other RTW-related questions.

#### Secondary outcomes

Secondary outcomes included self-reported health-related quality of life, impairments in mental functioning, overall job satisfaction, RTW self-efficacy, psychological symptom severity and work ability. Baseline measurements included socio-demographics, and characteristics related to health, psychosocial environment, job, treatment and labor market. A detailed overview of the measurement and computation of all outcomes can be found in Additional file 1.

### Randomization and blinding

Randomization was performed individually by an independent administrative working group not involved in the study. A computer-generated allocation sequence [32] used permuted block randomization with varying block sizes (4–10), stratified by study center. The online baseline assessment occurred before group assignment to minimize reporting and selection bias. Participants were randomized (1:1) according to their chronological order of baseline participation to either the intervention group (IG; undergoing RTW-PIA added to CAU), or the control group (CG, receiving CAU only). There was no blinding among participants, treatment providers, and evaluators, but an independent and blinded statistician performed the analyses.

### Statistical analysis

The performed analyses followed the study protocol [17] and a statistical analysis plan, preregistered before database closure (https://osf.io/fyhzt; version 1.2, 27.02.2025). Baseline characteristics were compared between groups using *t*- and χ^2^-tests to confirm randomization balance. All outcomes were analyzed on an intention-to-treat (ITT) basis, keeping randomized participants in their assigned groups regardless of adherence. Missing data were treated as missing at random (MAR), utilizing auxiliary variables for effect estimation. A total of 41 variables measured at T0 were included. A significance level of 5% and 95% confidence intervals (CI) were used for testing, and all analyses were performed using R 4.2.3.

The primary analysis compared the achievement of SRTW between the IG and CG, using OR from logistic regression. Further logistic regression was done to control for study center and other variables at T0 with significant differences between groups. The secondary analysis looked at the impact of RTW-PIA on changes in secondary outcomes over 24-months using multiple-group latent growth curve models (LGCM).

As prespecified in [17], we conducted sensitivity analyses in the per-protocol and complete-case populations (participants with data available at all measurement points), as well as in prespecified diagnostic subgroups. The per protocol population comprised participants who received the a priori-defined minimum RTW-PIA intervention dose (≥ 3/8 Module A sessions, ≥ 6/9 Module B sessions, and ≥ 1/3 Module C video conference sessions). Diagnostic subgroups were operationalized as the two largest diagnostic groups (depressive episode and recurrent depressive disorder), as the remaining diagnoses were too rare for separate analyses. In addition, we conducted prespecified exploratory moderator analyses in the ITT population, testing interaction effects between treatment group and all 41 baseline variables at the prespecified significance level of 5%. No adjustment for multiple comparisons was prespecified. Because these moderator analyses were not designed as confirmatory tests, we interpret their results as hypothesis-generating. Additional details on the statistical analyses are provided in Additional file 2.

To aid interpretation, we additionally report Benjamini-Hochberg false-discovery-rate (FDR) adjusted *q*-values for all interaction tests and compare the observed rate of significant tests with the 5% rate expected by chance. We additionally conducted two post hoc intervention exposure analyses within the IG, namely a comparison of participants who did versus did not receive the minimum RTW-PIA intervention dose and a dose-response analysis using the number of attended sessions.

## Results

### Baseline characteristics

The mean age of participants was approximately 43 years (SD = 11), with more females than males (*n* = 283 vs. *n* = 200), and more than 50% (*n* = 254) belonging to the lower gross household income groups (≤€2,999/month). Most of the participants in both groups were full-time, permanent employees of large companies. Prior to the study more than half of the participants in both groups had received day clinic care. The sample showed moderate depressive symptoms, low RTW self-efficacy, and poor work ability at baseline despite previous psychiatric treatment. A detailed summary of baseline characteristics is presented in Table 2. Some baseline characteristics achieved statistical significance between groups: the CG had a higher proportion of participants with migrant backgrounds (12.7% vs. 5.4%, *p* = 0.005) and reported unfavorable conditions more often regarding social support from both colleagues (*p* = 0.034) and supervisors (*p* = 0.003) and quality of leadership (*p* = 0.035).

**Table 2:** Participant characteristics at baseline (intention-to-treat-sample)

		RTW-PIA + CAU (*N* = 240)	CAU only (*N* = 244)	*p*
***Socio-demographic characteristics***				
**Gender** (female), n (%) ^1^		149 (62.1)	134 (54.9)	0.098
**Age** (years), mean ± SD		42.91 ± 11.06	42.25 ± 10.96	0.510
**Married/in a relationship** (yes), n (%)		117 (48.8)	113 (46.3)	0.591
**Cohabitation**, mean ± SD		2.19 ± 1.07	2.26 ± 1.29	0.487
**Person/s < 14 years living in household**, mean ± SD ^2^		1.39 ± 0.57	1.58 ± 0.58	0.931
**Highest school-leaving qualification**, n (%) ^3^				0.205
Finished school without degree		3 (1.3)	3 (1.2)	
Primary/secondary school-leaving certificate		18 (7.5)	22 (9.0)	
Intermediate / Secondary school-leaving certificate, polytechnical secondary school		71 (29.6)	81 (33.2)	
Technical college entrance qualification		38 (15.8)	39 (16.0)	
High school diploma (with/without vocational training) (university entrance qualification)		107 (44.6)	94 (38.5)	
**Highest educational/vocational qualification**, n (%) ^4^				0.163
No educational / vocational qualification		9 (3.8)	11 (4.5)	
Completion of vocational training (apprenticeship)		86 (35.8)	100 (41.0)	
Completion of vocational training (vocational or commercial school)		30 (12.5)	37 (15.2)	
Degree from a technical college, master/technical school, vocational/technical academy		25 (10.4)	22 (9.0)	
University of Applied Sciences degree		21 (8.8)	18 (7.4)	
College [of higher education] / University degree		60 (25.0)	51 (20.9)	
**Migrant background** (yes), n (%)		13 (5.4)	31 (12.7)	**0.005**
**Socio-economic status index** [range 3–21], mean ± SD ^b, 5^		13.01 ± 3.21	12.80 ± 3.01	0.609
**Monthly gross household income**, n (%) ^4^				0.316
< €1,000		2 (0.8)	5 (2.0)	
€1,000–1,999		42 (17.5)	41 (16.8)	
€2,000–2,999		81 (33.8)	83 (34.0)	
€3,000–3,999		61 (25.4)	40 (16.4)	
€4,000–4,999		24 (10.0)	31 (12.7)	
> €5,000		16 (6.7)	26 (10.7)	
**Application for reduced earning capacity pension** (yes), n (%)		27 (11.3)	28 (11.5)	0.938
**Health insurance type**, n (%)				
Public		224 (93.3)	232 (95.1)	0.409
Private		16 (6.7)	12 (4.9)	
***Health characteristics***				
**Primary clinical ICD-10 diagnosis**, n (%)				0.483
Depressive episode (F32)		67 (27.9)	83 (34.0)	
Recurrent depressive disorder (F33)		138 (57.5)	125 (51.2)	
Phobic anxiety disorders (F40)		18 (7.5)	20 (8.2)	
Other		17 (7.1)	16 (6.6)	
**Sickness absence days during last 12 months**, mean ± SD		134.60 ± 94.62	140.53 ± 88.16	0.474
**Work ability** [range 0–10], mean ± SD ^b^		4.02 ± 2.10	3.73 ± 2.18	0.140
**RTW self-efficacy** [range 1–6], mean ± SD ^b^		3.36 ± 1.05	3.30 ± 1.09	0.504
**RTW expectation** (positive), n (%)		180 (75.0)	179 (73.4)	0.680
**Recognized disability degree** (yes), n (%)		42 (17.5)	45 (18.4)	0.787
**Impairments in mental functioning** [range 1–5], mean ± SD ^6, a^		2.66 ± 0.64	2.64 ± 0.68	0.743
**Psychological symptom severity** [range 0–72], mean ± SD ^a^		20.90 ± 12.49	21.43 ± 12.29	0.637
**Depressive symptoms** [range 0–27], mean ± SD ^a^		11.89 ± 5.69	12.44 ± 5.68	0.282
**Health-related quality of life** [range 20–99], mean ± SD ^a^		62.27 ± 11.98	61.51 ± 12.60	0.432
***Psychosocial environmental characteristics***				
**Interpersonal trust** [range 3–15], mean ± SD ^b^		9.47 ± 2.60	9.31 ± 2.71	0.497
**General social support** [range 6–30], mean ± SD ^b^		22.24 ± 5.22	21.88 ± 5.46	0.452
***Job characteristics***				
**Psychosocial work climate** [range 1–5], mean ± SD ^b^		2.28 ± 0.90	2.24 ± 0.88	0.653
**COPSOQ measures**				
* Demands at work* [range 0-100], mean ± SD	*Quantitative* **^,a^	60.47 ± 23.33	60.04 ± 23.66	0.841
	*Emotional* **^,a^	55.05 ± 32.96	52.00 ± 32.18	0.302
	*Work pace* **^,a^	65.36 ± 21.85	68.39 ± 21.68	0.126
	*Work Privacy Conflicts* **^,a^	56.61 ± 23.90	59.17 ± 24.05	0.240
	*Dissolution* **^,a^	41.30 ± 28.46	41.50 ± 29.17	0.941
* Work organization and job content* [range 0-100], mean ± SD	*Influence at work* **^,b^	42.29 ± 21.47	39.52 ± 21.20	0.152
	*Degrees of freedom (breaks/holidays)* **^,b^	59.27 ± 25.65	59.63 ± 26.32	0.879
* Interpersonal relations and leadership* [range 0-100], mean ± SD	*Social support (colleagues)* **^,b^	57.89 ± 24.01	53.17 ± 25.28	**0.033**
	*Social support (supervisor)* **^,b^	54.60 ± 26.84	46.91 ± 28.56	**0.003**
	*Quality of Leadership* **^,b^	43.18 ± 23.63	38.62 ± 23.49	**0.036**
* Additional factors* [range 0-100], mean ± SD	*Work environment/physical demands* *^,a^	29.55 ± 22.85	33.18 ± 24.60	0.093
	*Insecurity over working conditions* **^,a^	29.44 ± 26.00	33.91 ± 26.10	0.059
* Impacts* [range 0-100], mean ± SD	*Overall job satisfaction* **^,b^	41.15 ± 25.10	40.88 ± 25.79	0.909
***Treatment characteristics***				
**Study center** (place of intervention delivery), n (%)				0.997
Hamburg		71 (29.6)	72 (29.5)	
Hanover		67 (27.9)	67 (27.5)	
Rinteln		49 (20.4)	48 (19.7)	
Berlin		27 (11.3)	28 (11.5)	
Bad Berleburg		26 (10.8)	29 (11.9)	
**Type of treatment** (received prior to study participation), n (%)				0.270
Inpatient treatment (psychiatric hospital)		53 (22.1)	45 (18.4)	
Day clinic (psychiatric day hospital)		136 (56.7)	131 (53.7)	
Psychiatric outpatient treatment		43 (17.6)	57 (23.4)	
***Labor market characteristic***				
**Employment contract** (permanent), n (%)		223 (92.9)	233 (95.5)	0.223

M = mean; SD = standard deviation; sums of numbers and percentages in columns may not add up to the total value due to rounded or missing values; values in bold indicate *p* < 0.05. The *p*-value is based on simple logistic regressions predicting group membership in intervention group (IG) vs. control group (CG) and using 200 imputed datasets to deal with missing values (see Additional file 1). Ordinal variables (highest school-leaving qualification, highest educational and vocational qualification, monthly gross household income) are displayed by category for descriptive purposes. The *p*-values are based on models treating these variables as numerical (see Additional file 1). Higher values indicate higher educational attainment and higher income, respectively

^1^* N* = 1 diverse, value was set to missing; ^2^* N* = 333 were asked the question because they had > 1 person in their household; *N* = 239 with *N* = 0 persons in the household < 14 years of age were excluded; ^3^* N* = 8 with other school qualifications [*N* = 3 in IG; *N* = 5 in CG]; ^4^* N* = 14 with other training qualifications [*N* = 9 in IG; *N* = 5 in CG]; ^5^* N* = 32 did not provide any information on monthly gross income [*N* = 14 in IG; *N* = 18 in CG]; ^6^* N* = 3 missing [*N* = 0 in IG; *N* = 3 in CG]; ^a^ Higher values = negative/undesirable; ^b^ Higher values = positive/desirable; * Intensity (ranging from 0 = “to a very large extent” to 100 = “to a very small extent”); ** Frequency (ranging from 0 = “always” to 100 = “never/hardly ever”)

### Primary analysis

RTW-PIA did not increase the likelihood of achieving SRTW on average (OR = 1.38 [95% CI 0.89 to 2.12] *p* = 0.148) as compared to CAU only. The estimated conditional probabilities for achieving SRTW were 60.4% in the CG and 67.7% in the IG. After adjustment for confounders and stratification by clinic location, the effect size became slightly smaller (OR = 1.23 [95% CI 0.77 to 1.95] *p* = 0.382).

Of the participants with a determinable SRTW status, *n* = 119 of 175 (68.0%) in the IG and *n* = 102 of 163 (62.6%) in the CG achieved SRTW. More details are provided in Additional file 3 (Figure S1, Tables S1 and S2).

Details of the analyses underlying the results reported below are also provided in Additional file 3.

### Analysis of secondary outcomes

**Fig. 2:**
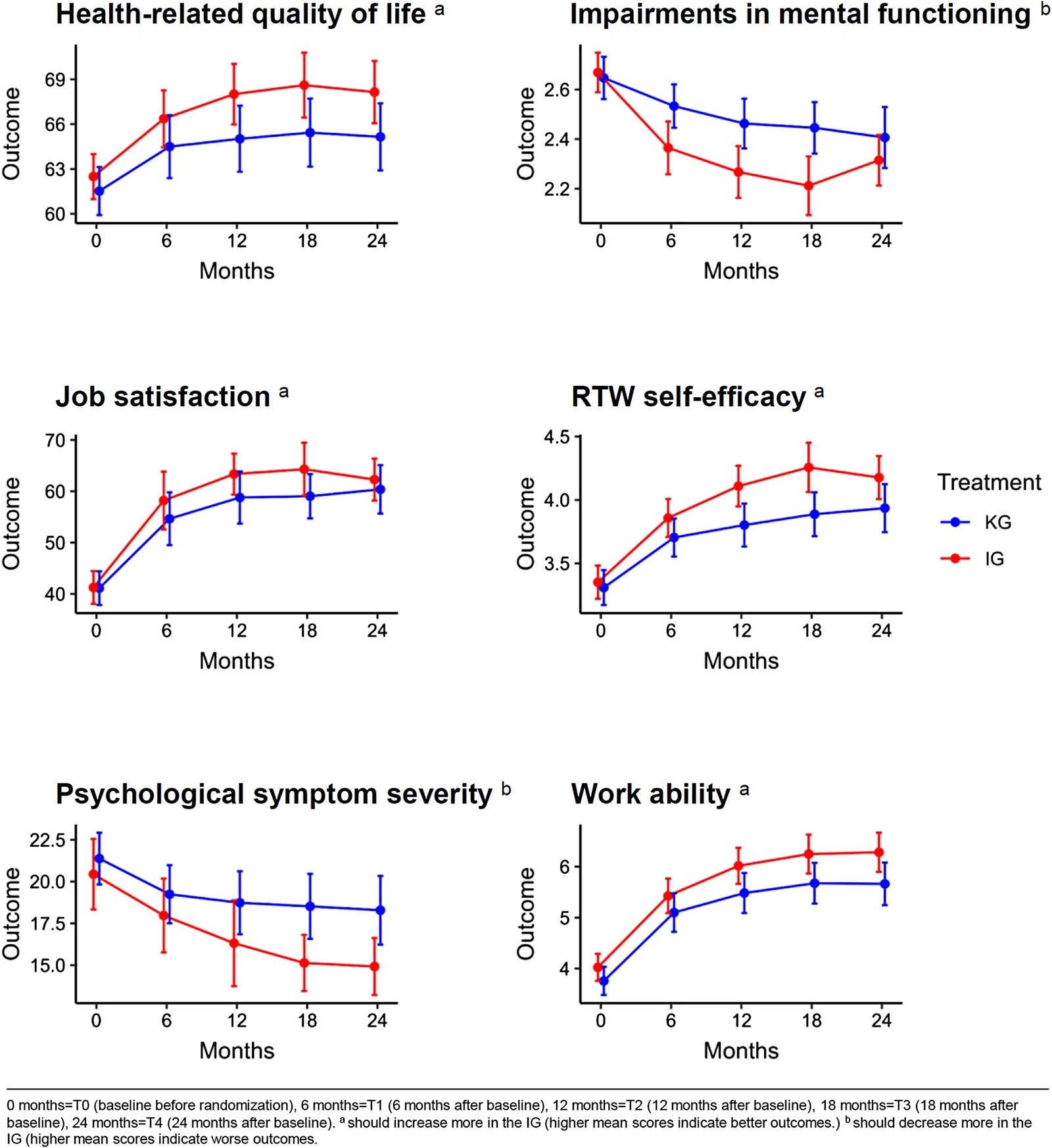
Model-implied mean trajectories of secondary outcome variables in ITT sample

The LGCM indicated non-significant differences in the trajectories of secondary outcomes over time (Fig. 2). Across all four post-baseline assessments (T1-T4), the IG generally showed more favorable mean outcome levels than the CG, and the effects of RTW-PIA were in the expected direction. In the IG, health-related quality of life (*d* = 0.124), job satisfaction (*d* = 0.066), RTW self-efficacy (*d* = 0.149), and work ability (*d* = 0.129) showed slight improvements, while impairments in mental functioning (*d*= -0.147) and psychological symptom severity (*d*= -0.170) decreased slightly more than in the CG. However, none of the estimated group differences achieved statistical significance (*p* ≥ 0.079) (Additional file 3, Tables S3 and S4).

### Delivery of intervention

The minimum intervention dose of Module A was achieved by 86% (*n* = 207) in the IG, significantly more than that of Module B (*n* = 116, 48.0%) or Module C (*n* = 80, 33.0%). Taken together, the overall minimum intervention dose of RTW-PIA was delivered to 24.6% (*n* = 59) of all IG-participants.

### Moderator analyses

The effect of RTW-PIA on SRTW achievement varied by the level of baseline variables, with four out of 41 tests (9.8%) yielding *p* < 0.05, compared with a chance expectation of 5.0% (Additional file 3, Table S6). In the unadjusted analyses, the treatment effect of RTW-PIA was moderated by recognized disability degree (IG: *n* = 42 with vs. *n* = 198 without; CG: *n* = 45 vs. *n* = 199), social support from colleagues, social support from supervisors, and type of health insurance (IG: *n* = 16 privately vs. *n* = 224 publicly insured; CG: *n* = 12 vs. *n* = 232; subgroup sizes in Additional file 3, Table S7). However, none of these interaction effects remained statistically significant after FDR adjustment (all *q* ≥ 0.425; Additional file 3, Table S6).

The impact of RTW-PIA showed significant differences between IG participants with and without a recognized disability degree (*p* = 0.025); those in the IG without a recognized disability degree had a 1.73 times higher likelihood of achieving SRTW than the CG (*p* = 0.024). We also found that the effect of RTW-PIA varied depending on health insurance status (*p* = 0.031), but the effect within subgroups did not achieve statistical significance (publicly insured: OR = 1.50, *p* = 0.072; privately insured: OR = 0.16, *p* = 0.113) (Additional file 3, Table S8).

In terms of social support from colleagues, the effect of RTW-PIA differed significantly among support levels (*p* = 0.033). IG participants who reported low social support from colleagues (-1 SD) were 2.08 times more likely to achieve a SRTW compared to the CG (*p* = 0.015). The effect of RTW-PIA also proved to be dependent on the support levels from their supervisors (*p* = 0.047). Those in the IG who reported low social support from supervisors were 1.97 times more likely to achieve a SRTW compared to those in the CG (*p* = 0.026) (Additional file 3, Table S9).

For the secondary outcomes, evidence of moderation was likewise limited. Of 226 interaction tests, only 16 (7.1%) were significant, barely above the expected random value of 5.0%. The most relevant moderators were the type of health insurance and diagnosis of a recurrent depressive disorder, both of which significantly moderated the treatment effect in three different secondary outcome variables. Specifically, RTW-PIA was significantly more effective in terms of mental functioning impairments, psychological symptom severity, and work ability in individuals diagnosed with recurrent depressive disorders than in individuals with other diagnoses (*p* ≤ 0.016).

After FDR adjustment, three interaction effects remained statistically significant: baseline RTW self-efficacy (*q* = 0.042) and baseline work ability (*q* = 0.048) moderated the treatment effect on mental functioning impairments, and a diagnosis of a recurrent depressive disorder moderated the treatment effect on work ability (*q* = 0.017). The latter remained significant after Bonferroni adjustment (Additional file 3, Table S10).

### Sensitivity analyses

Table 3 presents a summary of results across all sensitivity analyses of the secondary outcomes (per-protocol, complete-case, and diagnostic subgroup analyses). Among IG-participants receiving the minimum predefined RTW-PIA intervention dose (*n* = 59) and those responding to all data assessment time points (T0-T4) (*n* = 116), the effect of RTW-PIA on SRTW was not significant after adjustment for confounders and stratification by clinic location. Regarding changes in secondary outcomes, no significant group differences were observed (per-protocol: *p* ≥ 0.139; complete-case *p* ≥ 0.073). Participants responding at all five time points (*n* = 116) were on average healthier and socioeconomically more advantaged than those lost to at least one follow-up (*n* = 368), differing on 21 of 41 baseline characteristics (Additional file 3, Table S11).

**Table 3:** Summary of sensitivity analyses and post hoc analysis of the primary and secondary outcomes

			Estimated difference of total change in secondary Outcomes					
		Primary Outcome	Health-related quality of life ^d^	Impairments in mental functioning ^c^	Overall job satisfaction ^d^	RTW self-efficacy ^d^	Psychological symptom severity ^c^	Work ability ^d^
Sensitivity analyses	*n* IG/CG or other	OR [95% CI], *p*^a^	Between-group effect size Cohen´s *d*, *p*^b^					
Per-protocol ^1^	59/244	1.62 [0.75, 3.51], 0.224	0.103, 0.319	-0.195, 0.139	0.153, 0.377	0.192, 0.171	-0.101, 0.692	0.162, 0.208
Complete-case (T0-T4) ^2^	60/56	0.87 [0.16, 4.72], 0.871	0.237, 0.073	-0.274, 0.091	0.122, 0.582	0.260, 0.227	-0.102, 0.713	0.401, 0.087
***Diagnostic subgroups***								
Employees diagnosed with a depressive episode	67/83	1.21 [0.49, 2.98], 0.676	0.012, 0.927	0.082, 0.629	0.406, 0.048*	-0.080, 0.647	0.109, 0.466	-0.088, 0.617
Employees diagnosed with a recurrent depressive disorder	138/125	1.60 [0.86, 2.97], 0.141	0.305, < 0.001**	-0.346, 0.017*	0.105, 0.629	0.306, 0.048*	-0.350, 0.012*	0.402, 0.005*
**Post hoc analysis**								
Dose comparison within IG ^3^	59/181	1.31 [0.56, 3.04], 0.532	-0.018, 0.881	-0.108, 0.474	-0.012, 0.950	0.035, 0.840	0.147, 0.545	0.020, 0.894

ª Adjusted odds ratio for SRTW (IG vs. CG). Baseline variables that differed between groups and were adjusted for in each analysis are reported in Additional file 3, Table S2 (ITT Balance), Table S14 (Per protocol), Table S15 (Complete-case), Table S16 (F32), Table S17 (F33)

^b^ Standardized as in the ITT-analysis (FIML-estimated SD at 24 months in the reference group)

^c^ Higher scores on impairments in mental functioning and psychological symptom severity indicate less favorable states; negative *d* therefore indicates greater improvement in the IG (for comparison, the ITT effect sizes were health-related quality of life: 0.124; impairments in mental functioning − 0.147; job satisfaction 0.066; RTW self-efficacy 0.149; psychological symptom severity − 0.170; work ability 0.129)

^d^ Higher scores indicate favorable states; positive *d* therefore indicates greater improvement in the IG

^1^ Participants who achieved minimum RTW-PIA intervention dose vs. CG (The a priori-defined minimum RTW-PIA intervention dose was achieved if participants received ≥ 3/8 Module A sessions, ≥ 6/9 Module B sessions, and ≥ 1/3 Module C video conference sessions)

^2^ Participants who responded at all five assessments (T0-T4). The comparison is IG vs. CG within this group

^3^ Dose comparison within the intervention group: participants who achieved the minimum RTW-PIA intervention dose vs. participants who did not

*p < 0.05; ** *p* < 0.001

Among employees diagnosed with a depressive episode (*n* = 150) and those diagnosed with a recurrent depressive disorder (*n* = 263), the effect of RTW-PIA on SRTW was not significant after adjustments. Regarding changes in secondary outcomes, those diagnosed with a depressive episode in the IG showed a small statistically significant improvement in overall job satisfaction compared to the CG (*p* = 0.048). Participants diagnosed with recurrent depressive disorders in the IG showed significant improvements in health-related quality of life (*p* < 0.001), RTW self-efficacy (*p* = 0.048), work ability (*p* = 0.005), and reduced impairments in both psychological symptom severity (*p* = 0.012) and mental functioning (*p* = 0.017).

### Post hoc intervention exposure analyses

In a post hoc comparison within the IG, participants receiving the minimum intervention dose (*n* = 59) did not differ significantly from those who did not (*n* = 181) in achieving SRTW (*p* = 0.532) nor on any secondary outcome (*p* ≥ 0.474) (see Table 3; Additional file 3, Tables S12 and S13). Likewise, a complementary dose-response analysis yielded no significant association between the number of attended sessions and SRTW (OR per additional session = 1.05 [95% CI 0.96 to 1.15] *p* = 0.252).

## Discussion

In light of the general absence of structured RTW aftercare for employees with mental disorders post-discharge from psychiatric inpatient or day clinics, we evaluated the effectiveness of an 18-month, intensified RTW clinical aftercare, termed “RTW-PIA”. This clinical aftercare was implemented for the first time in German psychiatric outpatient clinics alongside conventional outpatient care and was compared to CAU there. Our study aimed to determine whether RTW-PIA when added to CAU (*i*) enhances SRTW in employees with mental disorders, (*ii*) improves secondary outcomes, and (*iii*) whether baseline characteristics moderate its effects.

The results did not confirm our hypotheses. On average, RTW-PIA significantly enhanced neither SRTW nor any of the secondary outcomes, and the per-protocol and complete-case sensitivity analyses led to the same conclusion. Prespecified exploratory moderator analyses and diagnostic subgroup analyses suggested that individuals without a recognized disability degree, those reporting lower levels of perceived social support from colleagues or supervisors, and those diagnosed with recurrent depressive disorders may benefit more from RTW-PIA. Of these, only the moderation of the effect on work ability by recurrent depressive disorder remained significant after multiple-testing correction. Two further interactions also remained significant after FDR adjustment, namely the interactions of baseline RTW self efficacy and work ability with the effect on impairments in mental functioning.

The observation of a consistent descriptive pattern of slight improvements across all secondary outcomes in the ITT sample is noteworthy and aligns with the study hypotheses. The IG showed slightly superior improvements in health-related quality of life, job satisfaction, RTW self-efficacy, and work ability, as well as slightly reduced impairments in psychological symptom severity and mental functioning compared to the CG. Although the direction of effects consistently favored RTW-PIA, these results were generally not statistically significant. However, the assumptions underlying the RTW-PIA program theory may hold for specific participants. With the exception of job satisfaction, all secondary outcomes significantly improved among employees diagnosed with recurrent depressive disorders, who constituted the vast majority of the sample. Hence, these results suggest an opportunity for refinement and additional testing of RTW-PIA, rather than evidence of failure. The process evaluation and qualitative assessment undertaken alongside this RCT are currently prepared for publication and will show how RTW-PIA can be improved according to participant needs and treatment providers’ delivery experiences.

We assume that our study encountered difficulties owing to the diversity and heterogeneity of the recruited group. In contrast to most RTW intervention studies, which typically focus on employees with common mental disorders and the workplaces [33, 34], our research concentrated on a severely impacted cohort within the psychiatric outpatient clinics, often with prior hospitalization or day-care visits, and prolonged sickness absence averaging 135–140 days (SD ≈ 91). Adding to its clinical relevance in our study, many employees had additional comorbidities (recurrent depressive disorder as primary vs. sole diagnosis). Prior to randomization, patients still showed moderate depressive symptoms, low RTW self-efficacy, and poor work ability. All of this not only reflects the diverse needs of the sample [24] but also highlights the possible variability in treatment response and RTW outcomes.

### Targeting

The observed pattern of significant interaction effects suggests that the effectiveness of RTW-PIA was not uniform across participants. There is limited understanding how well RTW-PIA meets the needs of specific groups, emphasizing the importance of better alignment with participant characteristics, that is, targeting.

Perceived low support from colleagues or supervisors prior to RTW was associated with greater benefits from RTW-PIA, suggesting that treatment providers may have helped fill this gap in workplace support. For example, they may have alleviated feelings of isolation or assisted with adjusting workplace accommodations, thereby facilitating SRTW. The importance of social relations at work during the RTW process has been investigated closely [35] and identified as a ‘missing piece’ that complements formal reintegration frameworks [36]. Hence, the criterion of low social support for enhancing targeting in RTW-PIA requires more investigation.

The finding that disability status influenced the success of achieving SRTW could also be related to the availability of external support. Employees in the IG without a recognized disability degree had higher success rates than those with a recognized disability, even though both subgroups faced severe mental disorders. It is plausible that those in the CG who have a recognized disability already have access to external support such as legal protection or job security, which in turn may have reduced the additional effect of RTW-PIA.

These subgroup findings underline the importance of considering both individual factors (such as disability status) and contextual factors (such as social workplace support) when designing and targeting RTW interventions.

### Implementation

The findings show that about 25% of participants received the minimum RTW-PIA intervention dose. Adherence to the predefined minimum dose varied across modules (Module A: 86%, Module B: 48%, and Module C: 33%). These differences should be interpreted in light of the distinct characteristics of the modules. Exposure to Module A and C was tailored to participants’ needs; thus, lower intervention exposure does not necessarily reflect lower acceptance of the intervention. The observed variation in uptake across modules may reflect implementation barriers, preferences, or individual needs. This limited overall exposure to the intervention may have reduced the observable differences between RTW-PIA and CAU only, making it difficult to interpret the effects of RTW-PIA conclusively. However, a complementary dose-response analysis did not identify a significant association between the number of attended sessions and SRTW.

Nevertheless, the positive and relatively consistent pattern across secondary outcomes in the ITT sample and the observed significant subgroup findings with respect to the primary outcome suggest beneficial effects of RTW-PIA. The lack of an overall significant result in the primary outcome may reflect a combination of implementation challenges (e.g., little flexibility given the research setting and strict manual, contextual conditions, participant´s heterogeneous needs/”compliance”) rather than limitations in the program’s theory. The accompanying process evaluation and qualitative analysis will further explore these questions.

### Strengths and limitations

The study contributes significantly to the international field of RTW research in several ways: First, its 24-month study period enabled comprehensive data collection over time. Second, the study included a large and diverse sample of employees with various diagnosed mental disorders, reflecting actual clinical practice in Germany. Third, this RTW clinical aftercare was conducted and evaluated under ‘real world’ conditions, aligning with recommendations for RCTs which are the gold standard for evaluating effectiveness. Fourth, because of the rigorous study protocol, intervention diversity was controlled, which improved the quality of the evaluation design. Fifth, loss to follow-up was reduced by conducting brief telephone assessments to capture primary outcome data from participants with missing questionnaire data. Sixth, by considering over 40 auxiliary variables in estimating treatment effects, it was possible to strengthen the MAR assumption and reduce the distorting influence of systematic dropout processes.

While the study has several notable strengths, some limitations should also be considered. First, because there is no centrally recorded data on days of incapacity for work in Germany, the study relied on participants’ self-reports. To minimize potential recall bias regarding the primary outcome, participants were requested to continuously record their days of work incapacity on a list provided by the study centers. Second, the COVID-19 pandemic presented significant challenges for both participant recruitment (e.g., reduced hospital occupancy, personnel shortages) and the implementation of RTW-PIA in a clinical setting (e.g., organization of group sessions). Despite these challenges, the target sample size was achieved. Third, participants’ use of additional, uncontrolled treatments (e.g., outpatient psychotherapy, medical rehabilitation, extended hospital stays) may have affected the internal validity of the RCT. Fourth, the SRTW outcome was highly complex, relying on a variety of variables at different timepoints. To reduce inconsistencies in the primary outcome, each individual case was thoroughly evaluated by two or three independent evaluators. Fifth, the minimum intervention dose may have been overly rigid for an intervention designed to accommodate participants’ needs. Sixth, as these analyses are based solely on quantitative data, important contextual or experiential aspects of RTW-PIA may be overlooked by the IG and their treatment providers; complementary findings from the process and qualitative evaluation will address this and will be reported elsewhere. Seventh, the moderator analyses were prespecified in the study protocol and the statistical analysis plan as exploratory and not as confirmatory. The rate of significant interaction effects on the primary outcome (9.8%) only modestly exceeded the chance expectation of 5.0%, and none of these effects remained significant after FDR adjustment. Combined with limited power in several subgroups, these moderator and subgroup findings should be considered hypothesis-generating and require independent confirmation. Eighth, intervention uptake between participants receiving the minimum intervention dose and non-achievers was not randomized, and participants differed in baseline characteristics. Therefore, adherence-related analyses are susceptible to self-selection and do not allow causal interpretation. Lastly, the amount or intensity of CAU received by participants or additional services received beyond the assigned study conditions were not assessed. This limits our ability to understand how these differences might have affected observed outcomes.

### Implications for research and practice

To further evaluate the effectiveness of RTW-PIA, we propose a follow-up study with an adapted target group focusing on patients diagnosed with depressive disorders, without a recognized disability degree or with low social support at the workplace, while potentially reconsidering the hypothesized effect size. A process evaluation is currently elaborating the extent to which the intervention itself requires content updates, including opportunities for tailoring (i.e., greater flexibilization and personalization). This is because RTW trajectories seem highly individual and the time required might vary, for example, due to differences in the duration of sickness absence, the severity of symptoms, and workplace conditions. Further research is thus also required to get a better understanding of which RTW-PIA components work best for whom.

Some basic principles of this intensified RTW clinical aftercare could be applied for other countries, given the challenges experienced by employees with mental disorders, for example in long-term labor force participation and social inclusion, are universal [37]. RTW-PIA was primarily conducted in a specific German setting, which may not entirely match approaches in other countries where hospitalizing patients with mental disorders is rare, like in the Netherlands or Sweden [38]. However, Belgium and the Netherlands have developed hospital-based interventions to promote work participation for certain clinical groups (e.g., patients with diagnosed breast cancer [39] and those with diagnosed chronic diseases [40]). These international interventions may gain additional knowledge from RTW-PIA on how to implement and plan a patient´s SRTW process according to four phases (co-orientation, coordination, cooperation, and re-co-orientation) in a needs-based approach, regardless of diagnosis [13]. RTW-PIA´s aftercare approach may furthermore serve as an example for how to implement and combine specific intervention theory programs across several modules over a longer, more sustainable span.

Discussing work and RTW as well as providing RTW support in psychiatric settings within an aftercare concept remains relevant. Clinical partners of RTW-PIA plan to provide a revised guidebook for healthcare stakeholders, occupational health managers, and therapy practitioners.

People with mental disorders are a very heterogeneous group, for example in terms of their RTW needs, and the course of their illness. This implies that it will be difficult to help all people with one intervention, hence targeting the groups in need and tailoring to individuals seem necessary. Further, some individuals in this trial with severe mental illnesses may have required continued support, such as provided by the successful Individual Placement and Support/Job Coaching program [41]. Hence, a continuous RTW-PIA version for this group might be worth evaluating.

Lambreghts et al. [42] advise incorporating qualitative feasibility studies before full implementation to obtain essential insights into the factors influencing intervention uptake, engagement, and effectiveness. Our study did not incorporate such an assessment, emphasizing the need for possible population- and setting-specific adaptions of RTW-PIA and future RTW interventions.

## Conclusions

This RCT did not confirm the effectiveness of RTW-PIA. The intervention significantly improved neither SRTW nor any secondary outcome in the total sample, and this finding was robust across sensitivity analyses. Prespecified exploratory moderator analyses and diagnostic subgroup analyses pointed to three groups that may benefit more from RTW-PIA. These were individuals without a recognized disability degree, those reporting lower levels of perceived social support from colleagues or supervisors, and those diagnosed with recurrent depressive disorders. Among these subgroup findings, only the moderation of the effect on work ability by recurrent depressive disorder remained significant after multiple-testing correction. All subgroup findings require independent confirmation before informing subgroup-specific recommendations. Future studies are warranted to determine which RTW-PIA components work best for whom, as well as to assess the intervention´s overall benefits to society. Given the need to maintain employability and the prevailing demographic trends leading to a deficit of trained labor, it will be important in the future to give greater consideration to the issues of work and RTW in clinical practice, as envisioned in RTW-PIA.

## Supplementary Information

Below is the link to the electronic supplementary material.

**Supplementary Material 1**: A detailed overview of the measurement and computation of all outcomes.

**Supplementary Material 2**: Additional details on the statistical analyses.

**Supplementary Material 3**: Details of the analyses behind the statistics reported.

## Data Availability

Data supporting the findings of this study are available from the corresponding author (FS) upon reasonable request, following consultation within the RTW-PIA consortium. Access to the final pseudonymized trial dataset may be granted to qualified researchers, subject to data security requirements and data-sharing agreements. The data are not publicly available due to privacy restrictions related to research participants.

## Abbreviations

CAU: Care as Usual
CI: Confidence interval
CG: Control group
FDR: False-discovery-rate
IG: Intervention group
ITT: Intention-to-treat
LGCM: Latent growth curve models
MAR: Missing at random
OR: Odds Ratio
RCT: Randomized controlled trial
RTW: Return to work
RTW-PIA: Return to work-Psychiatrische Institutsambulanz [German, name of studied intervention]
SD: Standard deviation
SRTW: Sustainable return to work
