# Supplementary material for: Intensified return to work clinical aftercare for sick-listed employees with diagnosed mental disorders in Germany: effects of a multicentre randomized controlled trial
Source: BMC Psychiatry. 2026 Sep 24;26:754. doi: 10.1186/s12888-026-08639-0 (PMC13632429; doi:10.1186/s12888-026-08639-0)
Supplement: Supplementary file 1 [file 12888_2026_8639_MOESM1_ESM.pdf]

Additional file 1. A detailed overview of the measurement and computation of all outcomes

Primary outcome

The primary outcome was the employees' self-reported achievement of sustainable RTW (SRTW). SRTW was confirmed by reporting ≤ 6 weeks of contractually agreed working days missed out due to sickness absence within 12 months after full RTW, hence after completion of all gradual RTW measurements.

As outlined in the protocol [1], the measurement of the primary outcome is based on the following two items: **(I)** *RTW* and **(II)** *No relapse after RTW*.

(I) RTW

At T1, T2, T3 and T4, all employees received the following self-developed question: "*Have you meanwhile returned to work?*" with the answer options 'yes' and 'no'. Meanwhile refers to the period since the last completed survey. The selected answer (yes) confirms the employee´s **first RTW**. The returned employees will be asked to numerically enter the date of their first RTW ("*When was your first day back at work?*" (DD.MM.YYYY)).

The latter does not yet represent a full RTW, as employees in Germany are legally permitted to use gradual RTW measurements from their employers' Occupational Integration Management (OIM). Since 2004, German employers have been required to commence an OIM process for employees who have been absent due to illness for more than six weeks in a calendar year, either continuously or intermittently (source).

Gradual RTW is a frequent OIM measure in which sick-listed employees gradually resume work (e.g., beginning with 2 hours per day) under medical supervision while collecting 70% of their wage through social insurance. The gradual RTW process typically lasts four to eight weeks, but it can also last up to six months or, in some circumstances, twelve months, depending on the patient´s healing process. A gradual RTW involves multiple stakeholders, including physicians, occupational therapists, health insurance providers, and employers, and it may get funding from a variety of insurance sources.

Thus, in the instance of gradual RTW, the first day of RTW represented the first day of gradual RTW, whereas the following end of gradual RTW, e.g., the start of contractually agreed working days, represented the day of **full RTW**. The exact date of full RTW, as well as the periods of gradual RTW that had occurred, were assessed from the participants in order to identify the start of the 12-month period of the primary outcome.

(II) No relapse after RTW

To verify the primary outcome input, employees who self-confirmed their RTW for the first time at either T1, T2, T3 or T4 (see **(I)**) received the following question: “*After returning to work, were you sick at home or on sick leave for more than six weeks in total?*” with the answer options ‘yes’ and ‘no’. The selected answer ‘no’ indicated that employees did not relapse after their RTW and might achieve SRTW in the future.

The achievement of SRTW was assessed at either 12 (T2), 18 (T3) or 24 months (T4) after baseline, depending on the participants’ RTW process[1]. Study participants who confirmed at T2, T3, or T4 that their RTW was more than 12 months ago, received the following question: “*In the first 12 months after returning to work, did you stay home sick or report sick for a total of more than 6 weeks?*” with the answer options ‘yes’ and ‘no’. All participants who answered ‘No’ to this question had achieved the primary outcome, e.g., a SRTW.

In cases of missing questionnaire data, participants were contacted via brief telephone assessments to obtain primary outcome data.

Back up variables to verify the primary outcome input

Participants were asked at all four measurement time points after baseline (T1-T4) to numerically indicate the total number of sickness absence since the last online-survey (six months ago) via an updated version from the Trimbos and Institute of Medical

[1] The 12 months had to fall entirely within the study's observation period; however, it was irrelevant whether the person had previously attempted an unsuccessful RTW.

Technology Assessment Cost Questionnaire for Psychiatry (TiC-P) [2]. The exact limit of sickness absence days per person was based on their working days per week[2].

Verification of self-reported SRTW data

All n=60 cases that reported unsustainable RTW were checked for coherency with their other self-reported data following a four-eyes principle. One case was changed to unclear and one case to SRTW, the other n=58 cases were coherent.

Categorization of cases without self-reported SRTW data

Due to non-participation at some assessments and the questionnaire filters not all participants were directly asked if they achieved SRTW or not. All cases without self-reported SRTW data were categorized into three groups 1) SRTW achieved, 2) SRTW not achieved, 3) unclear, not enough information available to determine SRTW status. The categorization was based on the back up variables mentioned above and critically verified by a second person. If discrepancies of the two categorizations occurred, the case was discussed with a third person until consensus was reached. Out of the n=148 cases without self-reported SRTW data n=13 were categorized as "SRTW achieved", n=59 as "SRTW not achieved", and n=76 as "unknown".

Secondary outcomes

Health-related quality of life

Health-related quality of life was measured using the 20-item Assessment of Quality of Life-6D (AQoL-6D) scale, which consists of six domains (independent living, relationship, mental health, coping, pain, senses) [3]. The AQoL-6D has 4 to 6-point response scales, with higher values indicating a lower quality of life. At baseline (T0), the online survey included additional information for individuals who may still have been undergoing inpatient treatment ("*For some questions, please imagine that you are at home if you are currently in the hospital*"). As recommended [4], we applied an unweighted scoring algorithm that reverses the scoring across all dimensions. The final total AQoL-6D score ranged from 0-100, with higher scores indicating the respondent´s higher quality of life.

---

[2] e.g., for a 5-day week, more than 30 sickness absence days constituted the threshold beyond which a person did not achieve a SRTW. For a 4-day week, the threshold was more than 24 sickness absence days.

Impairments in mental functioning

Mental functioning impairments were assessed with 13 items from the mental domain belonging to the 39-item Norwegian Function Assessment Scale (NFAS) [5], which assesses the physical and mental functioning in the working life and in daily life. The 39-items are divided in physical domains (covering Walking/standing, Holding/picking up things, Lifting/carrying and sitting) and mental domains (covering Managing, Cooperation/communication and Senses).

The NFAS-mental domains have a 5-point response scale, ranging from 1 (*no difficulty*) to 5 (*could not do it*), with higher values indicating more impairments in mental functioning. In accordance with Jankowiak et al., a sixth response category item was added ('*inapplicable to me, task not performed*'), and coded as a missing value. At least half of the items had to be answered to calculate any particular NFAS domain.

In the study protocol [1], we mentioned that data will be collected from the 7-item domain "Managing" and 6-item domain "Cooperation/Communication", with mental functioning as a secondary outcome. In order to decide whether the two domain "Managing" and "Cooperation/Communication" (13 item) can be combined into one overarching scale for mental functioning or whether to report the mean domain scores separated, we conducted several factor analyses, using the item responses at baseline (T0).

While the fit for a confirmatory two-factor model was not acceptable (CFI = .789, RMSEA = .124, SRMR = .081), the fit could be improved by allowing for two correlations between residuals (CFI = .919, RMSEA = .078, SRMR = .052). In the latter model, the latent correlation of the two scales was .82, suggesting that the two scale scores are largely redundant. An exploratory factor analysis with bifactor rotation produced a similar fit (CFI = .935, RMSEA = .086, SRMR = .047). This model comprised a strong g factor on which all but one of the items loaded clearly positively (> .40). The g factor had an explained common variance of 67.4% and could be measured with the 13-item total score with a reliability of .78 (in the sense of Omega Hierarchical).

Based on the results, the total mean scores of the completed items from the two NFAS domains (Managing and Cooperation/Communication) were combined into one

overarching scale for mental functioning impairments and used for the main analysis. In addition to the analyses specified in the study protocol or statistical analysis plan, we performed selected exploratory analyses for this outcome by testing the effect of IG (vs. CG) on the change in the two NFAS subscales "Managing" and "Cooperation/Communication" (see **Additional file 3**, Table S5).

Overall job satisfaction

Overall job satisfaction was measured using the single-item question from the Copenhagen Psychosocial Questionnaire job satisfaction scale [6]: "When you consider your work situation as a whole, how satisfied are you with your job overall, taking all circumstances into account?". The single-question has a 5-point response scale with the response options: *very satisfied*, *satisfied*, *unsatisfied*, *very unsatisfied*, and *not relevant*. We removed the last response option and added '*partly*' as a neutral response option to not force participants to a positive or negative answer. The score ranged from 100 to 0, with higher values indicating higher job satisfaction.

RTW self-efficacy

RTW self-efficacy was measured using the 11-item RTW self-efficacy (RTW-SE) questionnaire [7]. The RTW-SE had a 6-point response scale, ranging from 1 (*strongly disagree*) to 6 (*strongly agree*). The total score for the RTW-SE was calculated from the mean of 11 items, with higher scores indicating higher perceived self-efficacy in relation to RTW.

Severity of mental illness

Severity of mental illness was measured using three 6-item scales (somatization, depression, anxiety) from the Brief Symptom Inventory-18 (BSI-18) [8]. The three subscales have a 5-point response scale, ranging from 0 (*not at all*) to 4 (*very strongly*). The total value of the BSI-18, the Global Severity Index, ranges from 0 to 72, with higher values indicating greater symptom severity. The Global Severity Index was used for the analysis.

Work ability

Work ability was measured using the single-item Work Ability Score: "If you rate your best ever work ability as 10 points, how many points would you give your current work

ability?". The single-question has an 11-point response scale from 0 (*completely unable to work*) to 10 (*lifetime´s best work ability*) [9].

Minimum intervention dose

Treatment providers recorded participants' attendance in the RTW-PIA intervention modules using a participant-related documentation form, which was subsequently forwarded to the evaluating bodies. Based on these records, a dichotomous variable for the achievement of minimum intervention dose was created: (0) 'No' versus (1) 'Yes'. The minimum intervention dose was defined as attending at least:

- 3 of 8 Module A sessions,
- 6 of 9 Module B sessions, and
- 1 of 3 video conference sessions within Module C.

Participants who met these criteria were classified as having achieved the minimum intervention dose.

Baseline Measurements

(1) Socio-demographic characteristics

Gender (female, male, non-binary; the response item non-binary was set to missing), **age** (numeric input (DD/MM/YYYY); age at baseline was computed by subtracting the birth year from the year of assessment), **married/in a partnership** (assessed from marital status variable which had the following items: married/in a partnership, single, divorced/widowed; based on this, we computed a new variable 'married/in a partnership' by dichotomising the responses into (0) 'No' versus (1) 'Yes'), and **health insurance type** (public/private) were recorded via single-item questions from the German study on Mental Health at Work (German: Studie zur Mentalen Gesundheit am Arbeitsplatz (S-MGA)) .

Cohabitation was measured with two single-item questions from the GEDA (German: Gesundheit in Deutschland aktuell) [10]. Only those employees who numerically entered the question "How many people live in your household, including yourself?" with ≥ 1, were asked to enter the number of household people younger than fourteen years numerically ("How many people in your household are under the age of 14?"). Based on this, we computed the variable '**Person/s < 14 years living in household**' as the

number of household members younger than 14 years. This count variable was used in all analyses and is presented as mean and standard deviation.

Highest school leaving qualification was measured with a single-item question from the S-MGA on a 6-point response scale, with the response options: (1) Finished school without degree, (2) Primary/secondary school leaving certificate, (3) Intermediate/Secondary school leaving certificate, polytechnical secondary school, (4) Technical college entrance qualification, (5) High school diploma (with/-out) vocational training) (university entrance qualification), (6) Other degree. Response option 6 was set to missing so that it can be interpreted as a numerical variable.

Highest educational/vocational qualification was measured with a single-item question from the S-MGA [11] on a 7-point response scale, with the response options: (1) Completion of vocational training (apprenticeship), (2) Completion of vocational training (vocational or commercial school), (3) Degree from a technical college, master/technical school, vocational/technical academy, (4) University of Applied Sciences degree, (5) College [of higher education] / University degree, (6) Other qualification, and (7) No educational / vocational qualification. Response option 6 was set to missing and response option 7 was moved to the beginning of the new computed 6-point response scale so that it can be interpreted as a numerical variable.

Migrant background was measured via several questions from a survey instrument for mapping the presence of a migrant status. For this, Schenk et al. [12] has established a basic set of indicators for mapping migrant status. Allocation to having a migration background occurred if confirming that either a) employees' parents were born in another country ("In which country was your mother/father born?" (Germany/another country)), b) the employee has not lived in Germany since birth ("In which country were you born?" (Germany/Other, please specify)) and that at least one of the employees' parents was born abroad ("In which country was your mother/father born?"; Germany/another country)), or c) the employees' mother tongue is not German ("Is German your mother tongue?" (Yes/No). Based on this, we computed a new variable 'Migrant background' by dichotomizing the responses into (0) 'No' versus (1) 'Yes'; if one of the scenarios a) to c) were once confirmed (see underlined variables), the participants were allocated to 'Yes'.

Socio economic status index was measured in line with [10]. The SES index was calculated as a total score based on the point values assigned to the individual dimensions of education, occupational status, and monthly gross household income. Since the three sub-scales are weighted equally in the calculation, the SES index can take values between 3.0 and 21.0 (low 3.0–7.9; medium 8.0–13.8; high 13.9–21.0).

Monthly gross household income was measured with a single-item question, slightly adapted from the TiC-P [2] on a 7-point response scale as follows: (1) < €1,000, (2) €1,000-1,999, (3) €2,000-2,999, (4) €3,000-3,999, (5) €4,000-4,999, (6) > €5,000, and (7) I don´t want to specify. Response option 7 was set to missing so that it can be interpreted as a numerical variable.

Application for reduced earning capacity pension was measured with the following self-developed question, adapted from the Screening Instrument Work and Occupation (German: Screening-Instrument zur Messung des Bedarfs an berufsbezogenen Behandlungsangeboten in der medizinischen Rehabilitation (SIBAR)) [13]: "Are you currently considering applying for a pension for health reasons (disability pension)?", The single item had the following response options: (1) Yes, (2) No, and (3) I have already submitted a pension application. Based on this, we computed a new variable by dichotomizing the inputs into (0) 'No' versus (1) 'Yes'; responses (1) and (3) were assigned to 'Yes'.

(2) Health characteristics

Primary clinical ICD-10 diagnosis was therapist-registered and pseudonymized, using the ICD-10 codes. The affective disorders F31-39 were also separately presented.

Sickness absence days during last 12 months was retrospectively measured with the following self-developed question, adapted by the TiC-P [2]: "How many workdays in total did you stay home sick or call in sick in total during the last twelve months? Please indicate the number of days of incapacity for work during the last twelve months." [numeric input].

RTW expectation was measured with a single-item question from the SIBAR [13] on a 7-point response scale as follows: (1) immediately, (2) within one month, (3) - three months, (4) - six months, (5) - nine months, (6) - one year or later, and (7) not at all anymore. The question was slightly adapted to the study-specific setting as follows: “Considering your current state of health, when do you expect to return to work after your discharge?”. In accordance with [14], we dichotomized the responses into ‘positive (≤ 3 months)’ versus ‘negative (> 3 months)’; responses (4) to (7) were assigned to ‘negative’.

Recognized disability degree was measured with the following self-developed question, adapted from SIBAR [13]: “Do you have a recognized degree of disability?”. The single item had the following response options: (1) Yes, degree of < 50 and equivalent, (2) Yes, degree of < 50 but not equivalent, (3) Yes, degree ≥ 50, and (4) No. We dichotomised the responses into ‘Yes’ versus ‘No’; responses (1) to (3) were assigned into ‘Yes’.

Depressive symptoms were retrospectively measured with the 9-item Patient Health Questionnaire [15] on a 4-point response scale, ranging from (0) Not at all to (3) Nearly every day. An example items is: “Over the last two weeks, how often have you been bothered by any of the following problems? 1. Little interest or pleasure in doing things.”. The sum score ranges from 0 to 27, with higher scores indicating more depressive symptoms.

(3) Psychosocial characteristics

Interpersonal trust was measured using the short scale interpersonal trust (KUSIV3) [16]. The KUSIV3 consists of three items on a 5-point response scale with 1 (strongly disagree) to 5 (strongly agree). The scale score ranges from 3 to 15, with higher scores indicating higher interpersonal trust.

General social support was measured with the short version of the social support questionnaire (F-SozU K-6) [17]. The F-SozU K-6 consists of 6 items with a 5-point response scale from 1 (strongly disagree) to 5 (strongly agree). The scale score ranges from 6 to 30, with higher scores indicating higher perceived general social support.

(4) Job characteristics

Psychosocial work climate was measured using the Psychosocial Work Climate-4 (source). The PSC-4 consists of 4 items with a 5-point response scale from 1 (strongly disagree) to 5 (strongly agree). In line with Dollard et al. [18], we constructed the mean score for the PSC scale by determining the mean across items when at least 2 items were answered in the scale. The scale score ranges from 1 to 5, with higher scores indicating a healthy psychosocial safety climate.

COPSOQ measures were used as retrospective questions on psychosocial working conditions in relation to the last job before their current sickness absence, derived and modified from the Copenhagen Psychosocial Questionnaire (COPSOQ). Due to space constraints, the research team opted to include only few items for each COPSOQ scale instead of multiple items. The items were measured on five response categories, capturing either intensity (ranging from 0 = 'to a very large extent' to 100 = 'to a very small extent') or frequency (ranging from 0 = 'always' to 100 = 'never/hardly ever') [19]. According to the coding of answers, the mean values of items and scales are always between 0 and 100 points. At least half of the items on a scale had to be answered, otherwise the scale was a missing item and mean item scores could not be calculated accordingly [20]. The internal consistency of the German COPSOQ has been shown to be good or even very good reliability in relation to Cronbach's α [20]. The following scales were used:

Quantitative demands were measured with two items (example item "How often do you not have time to complete all your work tasks?"), higher scores represent unfavourable conditions[3] [19].
Emotional demands were measured with two items (example item "Do you have to deal with other people's personal problems as part of your work?"), higher scores represent unfavourable conditions[3] [19].
Work Pace was measured with two items (example item "Do you have to work very fast?"), higher scores represent unfavourable conditions[3] [19].

[3] Frequency (ranging from 0 = 'always' to 100 = 'never/hardly ever').

Work Privacy Conflicts[3] were measured with four items (example item "The demands of my work interfere with my private and family life."), higher scores represent unfavourable conditions [20].

Dissolution[3] was measured with two items (example item "I take care of work-related tasks outside of my working time as well."), higher scores represent unfavourable conditions [20].

Influence at Work[3] was measured with three items (example item "Do you have a large degree of influence on the decisions concerning your work?"), higher scores represent favourable conditions [20].

Degrees of freedom (breaks/holiday)[3] was measured with two items (example item "Can you take holidays more or less when you wish?", higher scores represent unfavourable conditions [20].

Social support from colleagues[3] was measured with two items (example item "How often do you get help and support from your colleagues?"), higher scores represent favourable conditions [19].

Social support from supervisors[3] was measured with two items (example item "How often is your immediate supervisor willing to listen to your work problems when needed?"), higher scores represent favourable conditions [19].

Quality of Leadership[3] was measured with four items (example item "To what extent would you say that your immediate superior is good at solving conflicts?"), higher scores represent favourable conditions [19].

Work environment/physical demands[4] were measured with six items (example item "How often do you have to do heavy physical work, e.g. heavy lifting, carrying or lifting?"), higher scores represent unfavourable conditions [20].

Insecurity over working conditions[3] was measured with three items (example item "Are you worried about being transferred to another job against your will?"), higher scores represent favourable conditions [19].

(5) Treatment characteristics

Study center was assessed using one self-developed single-choice item: "What clinic are you currently being treated at?" with the following five answer options (1) Alexianer

[3] Frequency (ranging from 0 = 'always' to 100 = 'never/hardly ever').
[4] Intensity (ranging from 0 = 'to a very large extent' to 100 = 'to a very small extent').

St. Joseph-Klinikum Berlin-Weißensee, (2) Asklepios Klinikum Harburg, (3) Burghof-Klinik Rinteln, (4) Klinik Wittgenstein, (5) Medizinische Hochschule Hannover.

Type of treatment was measured with the following self-developed question: “Where does your current treatment take place?” on a 4-point response scale as follow: (1) Inpatient treatment, (2) Day clinic, and (3) Outpatient treatment (psychiatric outpatient clinic), and (4) other [free text field]. In some cases, the free text field entries were assigned to one of the respective responses (1) to (3), if applicable.

(6) Labour market characteristics

Employment contract was measured with the following question from S-MGA [11]: “What type of employment contract do you have?”, with the response options (1) permanent and (2) fixed-term.

Resources

1. Starke F, Sikora A, Stegmann R, Knebel L, Buntrock C, de Rijk A, et al. Evaluating a multimodal, clinical and work-directed intervention (RTW-PIA) to support sustainable return to work among employees with mental disorders: study protocol of a multicentre, randomised controlled trial. BMC Psychiatry. 2023; doi:10.1186/s12888-023-04753-5.
2. Bouwmans C, De Jong K, Timman R, Zijlstra-Vlasveld M, Van der Feltz-Cornelis C, Tan Swan S, et al. Feasibility, reliability and validity of a questionnaire on healthcare consumption and productivity loss in patients with a psychiatric disorder (TiC-P). BMC Health Serv Res. 2013; doi:10.1186/1472-6963-13-217.
3. Richardson JR, Peacock SJ, Hawthorne G, Iezzi A, Elsworth G, Day NA. Construction of the descriptive system for the Assessment of Quality of Life AQoL-6D utility instrument. Health Qual Life Outcomes. 2012; doi:10.1186/1477-7525-10-38.
4. AQOL Assessment of Quality of Life. Using AQoL. n.d. https://www.aqol.com.au/index.php/scoring-algorithms/112-6d/231-spss-6d-psychometric. Accessed March 4 2026.
5. Jankowiak S, Rose U, Kersten N. Application of the ICF based Norwegian function assessment scale to employees in Germany. J Occup Med Toxicol. 2018; doi:10.1186/s12995-017-0183-4.
6. Pejtersen JH, Kristensen TS, Borg V, Bjorner JB. The second version of the Copenhagen Psychosocial Questionnaire. Scand J Public Health. 2010; doi:10.1177/1403494809349858.
7. Lagerveld SE, Blonk RWB, Brenninkmeijer V, Schaufeli WB. Return to work among employees with mental health problems: Development and validation of a self-efficacy questionnaire. Work & Stress. 2010; doi:10.1080/02678373.2010.532644.
8. Spitzer C, Hammer S, Lowe B, Grabe HJ, Barnow S, Rose M, et al. Die Kurzform des Brief Symptom Inventory (BSI-18): erste Befunde zu den psychometrischen Kennwerten der deutschen Version. Fortschr Neurol Psychiatr. 2011; doi:10.1055/s-0031-1281602.
9. El Fassi M, Bocquet V, Majery N, Lair ML, Couffignal S, Mairiaux P. Work ability assessment in a worker population: comparison and determinants of Work Ability Index and Work Ability score. BMC Public Health. 2013; doi:10.1186/1471-2458-13-305.
10. Lampert T, Kroll L, Müters S, Stolzenberg H. Messung des sozioökonomischen Status in der Studie zur Gesundheit Erwachsener in Deutschland (DEGS1). 2013; doi:10.1007/s00103-012-1663-4.
11. Pattloch D, Rose U, Menze L, Schöllgen I, Burr F, Buchallik F, et al. Data documentation of the Scientific Use File of the Study on Mental Health at Work (S-MGA), Waves 1 and 2, Version 2. 1st ed. Dortmund: Bundesanstalt für Arbeitsschutz und Arbeitsmedizin; 2025.
12. Schenk L, Bau AM, Borde T, Butler J, Lampert T, Neuhauser H, et al. Mindestindikatorensatz zur Erfassung des Migrationsstatus. Bundesgesundheitsblatt Gesundheitsforschung Gesundheitsschutz. 2006; doi:10.1007/s00103-006-0018-4.
13. Bürger W, Deck R. SIBAR – ein kurzes Screening-Instrument zur Messung des Bedarfs an berufsbezogenen Behandlungsangeboten in der medizinischen Rehabilitation. Rehabilitation (Stuttg). 2009; doi:10.1055/s-0029-1231062.
14. Sikora A, Schneider G, Wegewitz U, Bultmann U. Employees Receiving Inpatient Treatment for Common Mental Disorders in Germany: Factors Associated with Time to First and Full Return to Work. J Occup Rehabil. 2022; doi:10.1007/s10926-021-09985-4.

15. Kroenke K, Spitzer RL, Williams JB. The PHQ-9: validity of a brief depression severity measure. J Gen Intern Med. 2001; doi:10.1046/j.1525-1497.2001.016009606.x.
16. Beierlein C, Kemper C, Kovaleva AJ, Rammstedt B. Kurzskala zur Messung des zwischenmenschlichen Vertrauens: die Kurzskala Interpersonales Vertrauen (KUSIV3) [German only]. Mannheim: GESIS - Leibniz-Institut für Sozialwissenschaften; 2014.
17. Lin M, Hirschfeld G, Margraf J. Brief form of the Perceived Social Support Questionnaire (F-SozU K-6): Validation, norms, and cross-cultural measurement invariance in the USA, Germany, Russia, and China. Psychol Assess. 2019; doi:10.1037/pas0000686.
18. Dollard M, Dormann C, Idris MA. Psychosocial Safety Climate: A New Work Stress Theory and Implications for Method. In: Dollard M, Dormann C, Awang Idris M, editors. Psychosocial Safety Climate: Springer; 2019. p. 3-30.
19. Burr H, Berthelsen H, Moncada S, Nubling M, Dupret E, Demiral Y, et al. The Third Version of the Copenhagen Psychosocial Questionnaire. Saf Health Work. 2019; doi:10.1016/j.shaw.2019.10.002.
20. Lincke HJ, Vomstein M, Lindner A, Nolle I, Haberle N, Haug A, et al. COPSOQ III in Germany: validation of a standard instrument to measure psychosocial factors at work. J Occup Med Toxicol. 2021; doi:10.1186/s12995-021-00331-1.
