# Supplementary material for: Intensified return to work clinical aftercare for sick-listed employees with diagnosed mental disorders in Germany: effects of a multicentre randomized controlled trial
Source: BMC Psychiatry. 2026 Sep 24;26:754. doi: 10.1186/s12888-026-08639-0 (PMC13632429; doi:10.1186/s12888-026-08639-0)
Supplement: Supplementary file 2 [file 12888_2026_8639_MOESM2_ESM.pdf]

Additional file 2. Additional details on the statistical analyses

Statistical analysis

All outcomes were analyzed on an intention-to-treat basis, where randomized participants were retained in their originally assigned groups regardless of adherence or dropout. Missing data for the outcomes were assumed to be missing at random (MAR, e.g., missing at random conditional on information from all variables included in the respective model). To make the assumption of MAR plausible, we took auxiliary variables into account when estimating the effects (using multiple imputation for the primary outcomes, and full information maximum likelihood with saturated covariates for the secondary outcomes).

We tested interaction effects with the 41 variables measured at T0 (baseline) to test whether the effect of the intervention group (vs. control group) depended on the level of certain variables. For the primary outcome variable, we added the respective variable and its interaction term with the treatment group as a predictor to the logistic regression. For the secondary outcome variables, we included the respective variable as a predictor of the latent intercept and slope in both treatment groups and tested whether the effect on the latent slope differed between the treatment groups.

For the **primary outcome analysis**, we took the auxiliary variables into account using multiple imputation. More specifically, we used a “fully conditional specification” approach as implemented in the R package mice. To this end, we specified an imputation model for the primary outcome variable with all 41 auxiliary variables as predictors and performed the imputation process separately in the intervention group and control group. The basis was a Bayesian logistic regression model with indirectly integrated LASSO (Least Absolute Shrinkage and Selection Operator) regularization. A total of 200 imputed data sets were generated through this process. To estimate the intervention effect, the achievement of sustainable return to work was predicted in the 200 imputed data sets based on the treatment group (intervention group vs. control group) using logistic regression, and the 200 regression weights were then aggregated.

For the **secondary outcome analysis**, we took the auxiliary variables into account in the estimation of latent growth curve models (LGCM). To reduce the number of

auxiliary variables, we used only those auxiliary variables for each LGCM that were significantly associated with both the level and missingness of the target variable at a specific measurement point in time ($p < .05$) in the intervention group or control group. Specifically, this amounted to 8 to 16 auxiliary variables in each case. These variables were taken into account in the estimation of the LGCM using full information maximum likelihood as saturated correlates based on the R packages lavaan and semTools.

The LGCMs were based on data from five time points (T0-T4) and specified as a so-called “latent basis model” for two groups. This model is a less restrictive variant of the linear LGCM, e.g., it contains a latent intercept for individual differences at the beginning and a latent slope for individual differences in change over the entire period, but can also map nonlinear forms of change. The residual variances were fixed to one parameter between the treatment groups and time points. The focal test referred to the difference in mean latent slopes between the treatment groups. To determine a standardized effect size in terms of *d*, we divided this difference by the estimated standard deviation of the target variables at T4 in the control group. To account for possible deviations from a normal distribution of the target variables, we used scaled test statistics and robust standard errors.

Beyond this, and as described in the manuscript, we carried out sensitivity analyses, prespecified exploratory moderator analyses, and post hoc analyses (Table 1).

Table 1: Overview of the sensitivity, prespecified exploratory, and post hoc analyses

**Analysis**	**Prespecified in**	**Label**
**1)** Per-protocol (minimum RTW-PIA intervention dose[1] vs. CG)	Study Protocol *	Sensitivity analysis
**2)** Complete-case (T0-T4)[2]	Study Protocol	Sensitivity analysis
**3)** Diagnostic subgroups (F32/F33)	Study Protocol (“e.g.”)	Sensitivity analysis
**4)** Moderator analyses (41 / 226 interaction tests)	Study Protocol + Statistical Analysis Plan **	Prespecified exploratory moderator analysis
**5)** Dose comparison within IG[3], dose-response	Not prespecified (at reviewers’ request)	Post hoc

[1] The a priori-defined minimum RTW-PIA intervention dose was achieved if participants received ≥3/8 Module A sessions, ≥6/9 Module B sessions, and ≥1/3 Module C video conference sessions.
[2] Participants who responded at all five assessments (T0-T4). The comparison is IG vs. CG within this group.
[3] Participants who achieved the minimum RTW-PIA intervention dose vs. participants who did not.
* Starke F, Sikora A, Stegmann R, et al. Evaluating a multimodal, clinical and work-directed intervention (RTW-PIA) to support sustainable return to work among employees with mental disorders: study protocol of a multicentre, randomised controlled trial. *BMC Psychiatry*. 2023;23(1):380. doi:10.1186/s12888-023-04753-5
** Statistical plan, preregistered before database closure: https://osf.io/fyhzt/overview (version 1.2, 27.02.2025)
