# Supplementary material for: Intensified return to work clinical aftercare for sick-listed employees with diagnosed mental disorders in Germany: effects of a multicentre randomized controlled trial
Source: BMC Psychiatry. 2026 Sep 24;26:754. doi: 10.1186/s12888-026-08639-0 (PMC13632429; doi:10.1186/s12888-026-08639-0)
Supplement: Supplementary file 3 [file 12888_2026_8639_MOESM3_ESM.pdf]

Additional file 3. Details of the analyses behind the statistics reported

Primary analysis

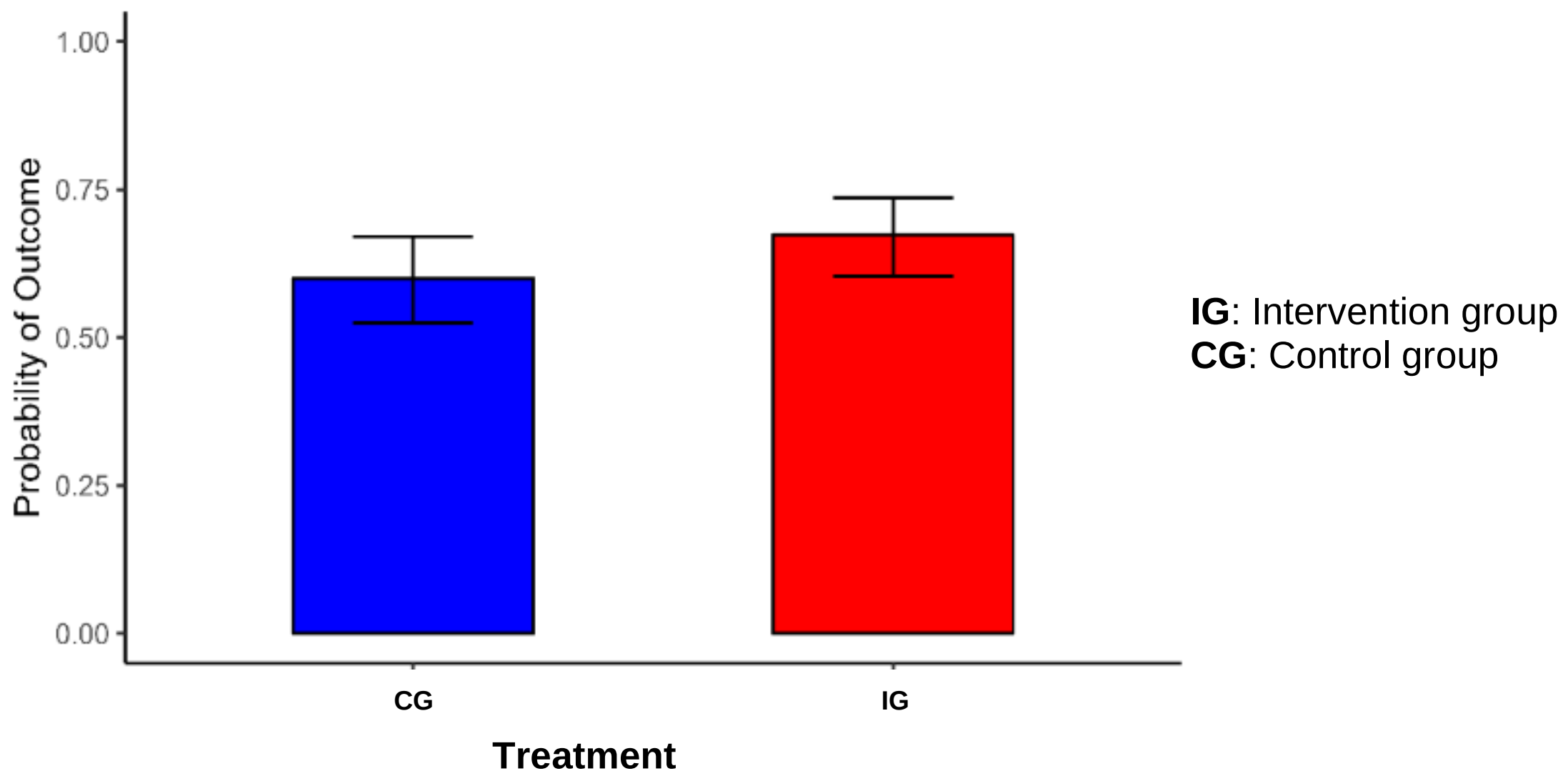

Figure S1 Estimated conditional probabilities for the primary outcome

Table S1 Observed distribution of the primary outcome by treatment group

	Control group (*n* = 244)	Intervention group (*n* = 240)
SRTW achieved, n (% of randomized)	102 (41.8)	119 (49.6)
SRTW not achieved, n (%)	61 (25.0)	56 (23.3)
SRTW status undetermined, n (%)[1]	81 (33.2)	65 (27.1)
SRTW achieved, % of determined cases	62.6	68.0

[1] Undetermined cases were treated as missing and multiply imputed (see Additional files 1 and 2).

Table S2 Assessment of Randomization Balance in the ITT Sample

**Baseline variables**	***p***
Health-related quality of life	0.432
Mental functioning impairments	0.743
Overall job satisfaction	0.909
RTW self-efficacy	0.504
Psychological symptom severity	0.637
Work ability	0.140
Depressive symptoms	0.282
Interpersonal trust	0.497
General social support	0.452
Age	0.510
Cohabitation	0.487
Person/s < 14 years living in household	0.931
Monthly gross household income	0.316
Socio-economic status index	0.609
Psychosocial work climate	0.653
Sickness absence days during last 12 months	0.474
Highest school-leaving qualification	0.205
Highest educational/vocational qualification	0.163
Emotional demands	0.302
Work pace	0.126
Quantitative demands	0.841
Physical demands	0.093
Influence at work	0.152
Degree of freedom (breaks/holidays)	0.879
Social support (colleagues)	**0.033**
Social support (supervisors)	**0.003**
Quality of Leadership	**0.036**
Work Privacy Conflicts	0.240
Dissolution	0.941
Insecurity over working conditions	0.059
Gender	0.098
Health insurance type	0.409
Employment contract	0.223
Migrant background	**0.005**
RTW expectation	0.680
Recognized disability degree	0.787
Married/in a relationship	0.591
Application for reduced earning capacity pension	0.938
Study center	0.997
Type of treatment	0.270
Primary clinical ICD-10 diagnosis	0.483

Values in bold indicate $p < .05$

Analysis of secondary outcomes

Table S3 Expected means and total estimated effects of RTW-PIA on secondary endpoints across full observation period (using LGCM)

		T0	T1	T2	T3	T4	Estimated difference of total change				
**Outcome**	Group	mean ± SE	mean ± SE	mean ± SE	mean ± SE	mean ± SE	**B** (est) (95% CI)	**SE**	**z**	**Between-group effect size Cohen´s *d***	***p***
**Psychological symptom severity** [range 0-72] [a]	IG	20.44 ± 1.077	17.97 ± 1.128	16.32 ± 1.307	15.13 ± 0.858	14.92 ± 0.870	- 2.430	1.384	-1.756	- 0.170	0.079
	CG	21.38 ± 0.789	19.24 ± 0.886	18.73 ± 0.960	18.52 ± 0.991	18.29 ± 1.047	(-5.142, 0.282)				
**Mental functioning impairments** [range 1-5] [a]	IG	2.66 ± 0.041	2.36 ± 0.054	2.27 ± 0.053	2.21 ± 0.060	2.31 ± 0.052	- 0.114	0.076	-1.495	- 0.147	0.135
	CG	2.65 ± 0.044	2.53 ± 0.045	2.46 ± 0.051	2.45 ± 0.053	2.41 ± 0.063	(-0.263, 0.035)				
**RTW self-efficacy** [range 1-6] [b]	IG	3.35 ± 0.067	3.86 ± 0.077	4.11 ± 0.082	4.26 ± 0.099	4.18 ± 0.087	0.199	0.134	1.485	0.149	0.138
	CG	3.31 ± 0.070	3.70 ± 0.076	3.80 ± 0.086	3.89 ± 0.088	3.94 ± 0.097	(-0.064, 0.461)				
**Overall job satisfaction** [range 0-100] [b]	IG	41.25 ± 1.631	58.19 ± 2.872	63.34 ± 2.037	64.29 ± 2.637	62.28 ± 2.084	1.775	3.536	0.502	0.066	0.616
	CG	41.12 ± 1.681	54.64 ± 2.629	58.78 ± 2.590	59.04 ± 2.199	60.37 ± 2.408	(-5.156, 8.705)				
**Health-related quality of life** [range 20-99] [b]	IG	62.49 ± 0.769	66.35 ± 0.973	68.01 ± 1.033	68.61 ± 1.108	68.14 ± 1.062	2.025	1.262	1.604	0.124	0.109
	CG	61.52 ± 0.819	64.50 ± 1.077	65.02 ± 1.128	65.43 ± 1.162	65.14 ± 1.144	(-0.449, 4.498)				
**Work ability** [range 0-10] [b]	IG	4.02 ± 0.135	5.43 ± 0.174	6.02± 0.181	6.25 ± 0.195	6.28 ± 0.196	0.352	0.284	1.243	0.129	0.214
	CG	3.76 ± 0.141	5.10 ± 0.193	5.48 ± 0.201	5.67 ± 0.204	5.66 ± 0.213	(-0.203, 0.908)				

IG intervention group; *CG* control group; *LGCM* Latent growth curve model. *SE* Standard Error.
T0=baseline [before randomization], T1=6 months after baseline, T2=12 months after baseline, T3=18 months after baseline, T4=24 months after baseline.
[a] Higher mean scores represent unfavorable conditions; [b] Higher mean scores represent favorable conditions.

Table S4 Descriptive statistics of the secondary outcomes between T1-T4 (observed cases)

Outcome	Group	T1<br>(N)<br>mean ± SD	T2<br>(N)<br>mean ± SD	T3<br>(N)<br>mean ± SD	T4<br>(N)<br>mean ± SD
**Psychological symptom severity** [range 0-72] [a]	IG	(178)<br>16.52 ± 12.44	(139)<br>14.08 ± 11.78	(127)<br>15.06 ± 12.63	(120)<br>14.63 ± 12.27
	CG	(152)<br>18.25 ± 12.96	(141)<br>19.31 ± 14.23	(128)<br>17.06 ± 13.62	(125)<br>17.06 ± 13.83
**Mental functioning impairments** [range 1-5] [a]	IG	(178)<br>2.34 ± 0.74	(139)<br>2.18 ± 0.72	(127)<br>2.14 ± 0.71	(120)<br>2.18 ± 0.70
	CG	(149)<br>2.50 ± 0.70	(138)<br>2.50 ± 0.74	(125)<br>2.39 ± 0.74	(124)<br>2.39 ± 0.79
**RTW self-efficacy** [range 1-6] [b]	IG	(178)<br>3.88 ± 1.12	(139)<br>4.15 ± 1.13	(127)<br>4.31 ± 1.21	(120)<br>4.28 ± 1.18
	CG	(153)<br>3.78 ± 1.06	(143)<br>3.82 ± 1.19	(128)<br>3.93 ± 1.26	(125)<br>3.98 ± 1.32
**Overall job satisfaction** [range 0-100] [b]	IG	(117)<br>60.68 ± 24.19	(110)<br>63.86 ± 23.61	(97)<br>64.95 ± 23.57	(94)<br>65.69 ± 23.47
	CG	(106)<br>61.08 ± 25.37	(103)<br>61.17 ± 26.83	(99)<br>61.62 ± 25.33	(91)<br>60.71 ± 26.65
**Health-related quality of life** [range 20-99] [b]	IG	(178)<br>67.76 ± 13.83	(139)<br>70.50 ± 14.24	(125)<br>69.39 ± 16.07	(120)<br>69.51 ± 15.31
	CG	(152)<br>65.51 ± 14.76	(141)<br>64.77 ± 15.07	(128)<br>66.92 ± 15.74	(125)<br>66.23 ± 15.62
**Work ability** [range 0-10] [b]	IG	(178)<br>5.53 ± 2.38	(139)<br>6.20 ± 2.30	(127)<br>6.15 ± 2.67	(120)<br>6.42 ± 2.44
	CG	(152)<br>5.38 ± 2.40	(142)<br>5.32 ± 2.61	(128)<br>5.70 ± 2.68	(125)<br>5.58 ± 2.67

IG intervention group; *CG* control group. *SD* Standard Deviation
T0=baseline [before randomization], T1=6 months after baseline, T2=12 months after baseline, T3=18 months after baseline, T4=24 months after baseline.
[a] Higher mean scores represent unfavorable conditions; [b] Higher mean scores represent favorable conditions.

Table S5 Expected means and estimated effects of RTW-PIA on the secondary endpoint of mental functioning impairments domains (“Managing” and “Cooperation/Communication”), across full observation period (using LGCM)

		T0	T1	T2	T3	T4	Estimated difference of total change				
**Outcome**	Group	mean ± SE	mean ± SE	mean ± SE	mean ± SE	mean ± SE	**B** (est) (95% CI)	**SE**	**z**	**Between-group effect size Cohen´s *d***	***p***
**Mental functioning impairments - “Managing” domain** [range 1-5] [a]	IG	2.98 ± 0.043	2.65 ± 0.058	2.49 ± 0.063	2.45 ± 0.069	2.57 ± 0.058	-0.081	0.090	-0.901	-0.093	0.368
	CG	2.98 ± 0.045	2.81 ± 0.050	2.73 ± 0.055	2.70 ± 0.060	2.65 ± 0.071	(-0.257, 0.095)				
**Mental functioning impairments - “Cooperation/Communication” domain** [range 1-5] [a]	IG	2.33 ± 0.048	2.07 ± 0.059	2.01 ± 0.055	1.96 ± 0.060	2.01 ± 0.054	-0.181	0.079	-2.283	-0.233	0.022
	CG	2.31 ± 0.049	2.24 ± 0.048	2.12 ± 0.057	2.18 ± 0.054	2.16 ± 0.062	(-0.337, -0.026)				

IG intervention group; *CG* control group; *LGCM* Latent growth curve model. *SE* Standard Error
T0=baseline [before randomization], T1=6 months after baseline, T2=12 months after baseline, T3=18 months after baseline, T4=24 months after baseline.
[a] Higher mean scores represent unfavorable conditions.

Table S6 Moderator analyses of the primary outcome in ITT-sample: unadjusted and multiplicity-adjusted *p*-values for all 41 baseline variables

		**Multiplicity-adjusted *p*-values for moderator analyses**	
**Baseline variables**	***p***	***q BH-FDR*** [1]	***p Bonferroni*** [2]
Health-related quality of life	0.560	0.877	1
Mental functioning impairments	0.703	0.900	1
Overall job satisfaction	0.745	0.900	1
RTW self-efficacy	0.538	0.877	1
Psychological symptom severity	0.529	0.877	1
Work ability	0.602	0.877	1
Depressive symptoms	0.816	0.911	1
Interpersonal trust	0.137	0.625	1
General social support	0.434	0.877	1
Age	0.822	0.911	1
Cohabitation	0.473	0.877	1
Person/s < 14 years living in household	0.450	0.877	1
Monthly gross household income	0.867	0.911	1
Socio-economic status index	0.219	0.747	1
Psychosocial work climate	0.393	0.877	1
Sickness absence days during last 12 months	0.621	0.877	1
Highest school-leaving qualification	0.642	0.877	1
Highest educational/vocational qualification	0.460	0.877	1
Emotional demands	0.348	0.877	1
Work pace	0.382	0.877	1
Quantitative demands	0.966	0.979	1
Physical demands	0.130	0.625	1
Influence at work	0.682	0.900	1
Degree of freedom (breaks/holidays)	0.096	0.625	1
Social support (colleagues)	**0.033**	0.425	1
Social support (supervisors)	**0.047**	0.425	1
Quality of Leadership	0.369	0.877	1
Work Privacy Conflicts	0.548	0.877	1
Dissolution	0.304	0.877	1
Insecurity over working conditions	0.132	0.625	1
Gender	0.746	0.900	1
Health insurance type	**0.031**	0.425	1
Employment contract	0.591	0.877	1
Migrant background	0.311	0.877	1
RTW expectation	0.866	0.911	1
Recognized disability degree	**0.025**	0.425	1
Married/in a relationship	0.153	0.625	1
Application for reduced earning capacity pension	0.183	0.682	1
Study center	0.979	0.979	1
Type of treatment	0.804	0.911	1
Primary clinical ICD-10 diagnosis	0.052	0.425	1

Values in bold indicate *p* < .05

[1] Benjamini–Hochberg false-discovery-rate *q*-values across all k = 41 tests

[2] Bonferroni-adjusted *p* values across all k = 41 tests

Moderator analyses

Table S7 Subgroup sizes and observed SRTW distribution for the significant moderators

Moderator / Level	Group	*n*	SRTW achieved	SRTW not achieved	SRTW undetermined	SRTW achieved, % of determined
**Health insurance type**						
Private	CG	12	10	1	1	90.9
	IG	16	8	6	2	57.1
Public	CG	232	92	60	80	60.5
	IG	224	111	50	63	68.9
**Recognized disability degree**						
No	CG	199	81	52	66	60.9
	IG	198	104	43	51	70.7
Yes	CG	45	21	9	15	70.0
	IG	42	15	13	14	53.6
**Social support** (*colleagues*) (M ± SD)	CG	243	/	/	/	53.1 ± 25.3
	IG	236	/	/	/	57.9 ± 24.0
**Social support** (*supervisors*) (M ± SD)	CG	243	/	/	/	46.9 ± 28.6
	IG	239	/	/	/	54.6 ± 26.8

IG = intervention group, CG = control group; SRTW = sustainable Return to Work.

Table S8 Significant interaction effects of baseline variables (categorical) with regard to the primary outcome

	**Intervention group** (IG) (*n*=240)	**Control group** (CG) (*n*=244)	SRTW achievement for IG with CG as reference group [a]	Interaction effect between groups
	Predicted probability (95% CI)		OR (95% CI), *p-value*	*p*
**Health insurance type**				.031
Private	0.604 (0.351, 0.811)	0.908 (0.558, 0.987)	0.155 (0.016, 1.549), *p*=.113	
Public	0.678 (0.606, 0.743)	0.584 (0.506, 0.658)	1.502 (0.963, 2.342), *p*=.072	
**Recognized disability degree**				.025
Yes	0.518 (0.353, 0.679)	0.684 (0.508, 0.820)	0.496 (0.182, 1.350), *p*=.170	
No	0.706 (0.631, 0.771)	0.581 (0.497, 0.660)	1.734 (1.075, 2.797), *p*=.024*****	

Data are imputed and based on ITT analyses; see Table 7 for subgroup sizes and observed SRTW distribution for the significant moderators

[a] Conditional odds ratio for achieving SRTW (IG vs. CG) at the respective level of the moderator

Table S9 Significant interaction effects of baseline variables (continuous) with regard to the primary outcome

	**Intervention group** (IG) (*n*=240)	**Control group** (CG) (*n*=244)	SRTW achievement for IG with CG as reference group [a]	Interaction effect between groups
	Predicted probability (95% CI)		OR (95% CI), *p-value*	*p*
**Social support** (*colleagues*) [1]				.033
Low [30.7]	0.667 (0.561, 0.758)	0.491 (0.393, 0.589)	2.076 (1.155, 3.732), *p*=.015*	
Average [50.5]	0.673 (0.603, 0.736)	0.618 (0.537, 0.692)	1.273 (0.809, 2.003), *p*=.292	
High [80.2]	0.679 (0.582, 0.763)	0.730 (0.618, 0.820)	0.781 (0.395, 1.543), *p*=.474	
**Social support** (*supervisors*) [1]				.047
Low [22.6]	0.673 (0.567, 0.765)	0.512 (0.413, 0.609)	1.969 (1.086, 3.571), *p*=.026*	
Average [50.7]	0.673 (0.603, 0.737)	0.618 (0.540, 0.691)	1.274 (0.818, 1.984), *p*=.285	
High [78.7]	0.673 (0.578, 0.756)	0.714 (0.607, 0.802)	0.824 (0.436, 1.558), *p*=.552	

[1] Scale ranges from 0-100 with higher mean scores representing favorable conditions.

[a] Conditional odds ratio for achieving SRTW (IG vs. CG) at the respective level of the moderator

Conditional treatment effects are reported at low (mean −1 SD), average (mean), and high (mean +1 SD) levels of the moderator. Data are imputed and based on ITT analyses; see Table 7 for subgroup sizes and observed SRTW distribution for the significant moderators

Table S10 Significant moderators of secondary outcomes in ITT sample (*p* < .05)

						Multiplicity-adjusted p-values for moderator analyses	
**Outcome**	**Moderator**	**B (est) (95% CI)**	**SE**	**z**	***p***	***q BH-FDR*** [1]	***p Bonferroni*** [2]
**Mental functioning impairments**	RTW self-efficacy	-0.220 (-0.342, -0.099)	0.062	-3.561	**<.001**	**0.042**	0.083
	Psychological symptom severity	0.016 (0.005, 0.026)	0.005	2.981	**0.003**	0.162	0.649
	Work ability	-0.103 (-0.162, -0.044)	0.030	-3.414	**0.001**	**0.048**	0.144
	Depressive symptoms	0.028 (0.006, 0.051)	0.012	2.451	**0.014**	0.395	1
	Emotional demands	0.006 (0.002, 0.010)	0.002	2.886	**0.004**	0.176	0.882
	Work pace	0.008 (0.002, 0.014)	0.003	2.575	**0.010**	0.366	1
	Gender	-0.291 (-0.571, -0.012)	0.143	-2.041	**0.041**	0.583	1
	Type of health insurance	0.564 (0.028, 1.099)	0.273	2.063	**0.039**	0.583	1
	Diagnosis of a recurrent depressive episode	-0.338 (-0.613, -0.064)	0.140	-2.415	**0.016**	0.395	1
**Psychological symptom severity**	Work pace	0.144 (0.021, 0.268)	0.063	2.292	**0.022**	0.413	1
	Diagnosis of a recurrent depressive episode	-6.084 (-10.794, -1.374)	2.403	-2.532	**0.011**	0.366	1
**Work ability**	Diagnosis of a recurrent depressive episode	2.051 (1.037, 3.066)	0.518	3.964	**<.001**	**0.017**	0.017
**Health-related quality of life**	Mental functioning impairments	-4.022 (-7.363, -0.681)	1.705	-2.360	**0.018**	0.404	1
	Gender	5.670 (0.905, 10.434)	2.431	2.332	**0.020**	0.404	1
	Type of health insurance	-7.901 (-15.437, -0.365)	3.845	-2.055	**0.040**	0.583	1
**RTW self-efficacy**	Type of health insurance	-1.008 (-1.891, -0.124)	0.451	-2.236	**0.025**	0.441	1

Values in bold indicate *p / q BH-FDR* < .05

[1] Benjamini–Hochberg false-discovery-rate *q*-values across all k = 226 interaction tests

[2] Bonferroni-adjusted *p* values across all k = 226 interaction tests

Table S11 Participant characteristics at baseline (participants who responded at all five assessments vs. those lost to at least one follow-up assessment)

		Completer (*n*=116)	Lost (*n*=368)	*p*
Socio-demographic characteristics				
**Gender** (female), n (%)		62 (53.9)	221 (60.1)	.249
**Age** (years), mean ± SD		43.59 ± 10.82	42.26 ± 11.06	.257
**Married/in a relationship** (yes), n (%)		59 (50.9)	171 (46.5)	.409
**Cohabitation**, mean ± SD		2.11 ± 1.12	2.26 ± 1.20	.232
**Person/s < 14 years living in household**, mean ± SD [1]		0.44 ± 0.76	0.41 ± 0.73	.801
**Highest school-leaving qualification**, mean ± SD [3]		4.17 ± 0.97	3.81 ± 1.11	**.001**
**Highest educational/vocational qualification**, mean ± SD [4]		3.02 ± 1.76	2.33 ± 1.65	**<.001**
**Migrant background** (yes), n (%)		4 (3.4)	40 (10.9)	**.008**
**Socio-economic status index** [range 3-21], mean ± SD [b, 5]		14.20 ± 2.89	12.50 ± 3.07	**<.001**
**Monthly gross household income**, mean ± SD [4]		3.87 ± 1.22	3.42 ± 1.22	**.001**
**Application for reduced earning capacity pension** (yes), n (%)		6 (5.2)	49 (13.3)	**.009**
**Health insurance type**, n (%)				**.023**
Public		104 (89.7)	352 (95.7)	
Private		12 (10.3)	16 (4.3)	
Health characteristics				
**Primary clinical ICD-10 diagnosis**, n (%)				.139
Depressive episode (F32)		43 (37.1)	107 (29.1)	
Recurrent depressive disorder (F33)		56 (48.3)	207 (56.2)	
Phobic anxiety disorders (F40)		12 (10.3)	26 (7.1)	
Other		5 (4.3)	28 (7.6)	
**Sickness absence days during last 12 months,** mean ± SD		106.26 ± 71.55	147.46 ± 94.75	**<.001**
**Work ability** [range 0-10], mean ± SD [b]		4.53 ± 2.07	3.67 ± 2.13	**<.001**
**RTW self-efficacy** [range 1-6], mean ± SD [b]		3.56 ± 0.93	3.25 ± 1.11	**.007**
**RTW expectation** (positive), n (%)		103 (88.8)	256 (69.6)	**<.001**
**Recognized disability degree** (yes), n (%)		17 (14.7)	70 (19.0)	.277
**Impairments in mental functioning** [range 1-5], mean ± SD [6, a]		2.47 ± 0.68	2.71 ± 0.64	**<.001**
**Psychological symptom severity** [range 0-72], mean ± SD [a]		17.48 ± 10.28	22.33 ± 12.76	**<.001**
**Depressive symptoms** [range 0-27], mean ± SD [a]		10.67 ± 5.30	12.64 ± 5.73	**.001**
**Health-related quality of life** [range 20-99], mean ± SD [a]		65.54 ± 11.03	60.74 ± 12.45	**<.001**
Psychosocial environmental characteristics				
**Interpersonal trust** [range 3-15], mean ± SD [b]		9.86 ± 2.56	9.24 ± 2.67	**.027**
**General social support** [range 6-30], mean ± SD [b]		22.68 ± 5.13	21.86 ± 5.39	.145
Job characteristics				
**Psychosocial work climate** [range 1-5], mean ± SD [b]		2.30 ± 0.76	2.25 ± 0.93	.644
**COPSOQ measures**				
Demands at work [range 0-100], mean ± SD	*Quantitative* **,a	60.13 ± 21.55	60.29 ± 24.08	.948
	Emotional **,a	49.25 ± 31.68	54.86 ± 32.78	.105
	Work pace **,a	65.84 ± 18.96	67.22 ± 22.63	.552
	Work Privacy Conflicts **,a	54.80 ± 24.66	58.88 ± 23.71	.110
	Dissolution **,a	43.10 ± 29.18	40.86 ± 28.68	.465

Work organization and job content [range 0-100], mean ± SD	*Influence at work* **,b	43.03 ± 18.91	40.22 ± 22.06	.216
	Degrees of freedom (breaks/holidays) **,b	62.72 ± 25.59	58.42 ± 26.03	.116
Interpersonal relations and leadership [range 0-100], mean ± SD	*Social support (colleagues)* **,b	58.11 ± 23.69	54.66 ± 25.05	.188
	Social support (supervisor) **,b	52.39 ± 25.05	50.20 ± 28.82	.429
	Quality of Leadership **,b	42.61 ± 22.21	40.35 ± 24.08	.372
Additional factors [range 0-100], mean ± SD	*Work environment/physical demands* *,a	24.32 ± 20.84	33.61 ± 24.26	**<.001**
	Insecurity over working conditions **,a	26.94 ± 26.08	33.20 ± 25.98	**.022**
Impacts [range 0-100], mean ± SD	*Overall job satisfaction* **,b	43.97 ± 23.69	40.08 ± 25.91	.150
Treatment characteristics				
**Study center** (place of intervention delivery), n (%)				**.002**
Hamburg		43 (37.1)	100 (27.2)	
Hanover		38 (32.8)	96 (26.1)	
Rinteln		10 (8.6)	87 (23.6)	
Berlin		14 (12.1)	41 (11.1)	
Bad Berleburg		11 (9.5)	44 (12.0)	
**Type of treatment** (received prior to study participation), n (%)				**.010**
Inpatient treatment (psychiatric hospital)		15 (13.4)	83 (23.5)	
Day clinic (psychiatric day hospital)		78 (69.6)	189 (53.5)	
Psychiatric outpatient treatment		19 (17.0)	81 (22.9)	
Labor market characteristic				
**Employment contract** (permanent), n (%)		116 (100.0)	340 (92.4)	**<.001**

M = mean; SD = standard deviation; sums of numbers and percentages in columns may not add up to the total value due to rounded or missing values; values in bold indicate $p < .05$. The p-value is based on simple logistic regressions predicting completer status (responded at all five assessments (T0-T4) vs. lost to at least one follow-up assessment (T1-T4) and using 200 imputed datasets to deal with missing values (see Additional file 1). Descriptive statistics are based on observed cases.

The three ordinal variables (highest school-leaving qualification, highest educational and vocational qualification, monthly gross household income) were treated as numerical (see Additional file 1). Higher values indicate higher educational attainment and higher income, respectively

[a] Higher values = negative/undesirable; [b] higher values = positive/desirable

* Intensity (ranging from 0 = “to a very large extent” to 100 = “to a very small extent”); ** Frequency (ranging from 0 = “always” to 100 = “never/hardly ever”).

[c] Estimates for employment contract should be interpreted with caution due to quasi-complete separation, as there were no fixed-term contracts in this group

Table S12 Total change difference in secondary outcomes between those having received the minimum RTW-PIA intervention dose (*n*=59) vs. those who did not (*n*=181), within the IG

	Estimated difference of total change				
**Outcome**	**B** (est) (95% CI)	**SE**	**z**	**Between-group effect size Cohen´s *d***	***p***
**Psychological symptom severity** [range 0-72] [a]	1.694 (-3.793, 7.180)	2.799	0.605	0.147	0.545
**Mental functioning impairments** [range 1-5] [a]	-0.074 (-0.275, 0.128)	0.103	-0.717	-0.108	0.474
**RTW self-efficacy** [range 1-6] [b]	0.039 (-0.340, 0.418)	0.193	0.202	0.035	0.840
**Overall job satisfaction** [range 0-100] [b]	-0.317 (-10.198, 9.563)	5.041	-0.063	-0.012	0.950
**Health-related quality of life** [range 20-99] [b]	-0.273 (-3.851, 3.305)	1.826	-0.149	-0.018	0.881
**Work ability** [range 0-10] [b]	0.049 (-0.676, 0.775)	0.370	0.133	0.020	0.894

[a] Higher mean scores represent unfavorable conditions; [b] Higher mean scores represent favorable conditions.

Table S13 Baseline differences between IG participants who received the minimum RTW-PIA intervention dose (*n*=59) and those who did not (*n* = 181)

**Variable**	**AUC**	**SE**	***p***	**Group**		***n***	**mean ± SD / %**
**Health-related quality of life** [range 20-99] [a]	0.585	0.045	**0.040**	noMD		181	61.44 ± 11.26
				MD		59	65.07 ± 13.33
**Impairments in mental functioning** [range 1-5] [a]	0.576	0.046	**0.043**	noMD		181	2.71 ± 0.61
				MD		59	2.52 ± 0.70
**General social support** [range 6-30]	0.65	0.040	**0.001**	noMD		181	21.6 ± 5.26
				MD		59	24.2 ± 4.59
**Cohabitation**	0.634	0.040	**0.002**	noMD		181	2.3 ± 1.08
				MD		59	1.83 ± 0.99
**Person/s < 14 years living in household**	0.590	0.049	**0.045**	noMD		138	0.43 ± 0.72
				MD		32	0.28 ± 0.58
**Monthly gross household income**	0.612	0.04	**0.019**	noMD		171	3.38 ± 1.17
				MD		55	3.84 ± 1.01
**Socio-economic status index** [range 3-21],	0.636	0.044	**0.001**	noMD		171	12.61 ± 3.16
				MD		55	14.26 ± 3.07
**Highest school-leaving qualification**	0.590	0.040	**0.031**	noMD		179	3.88 ± 1.08
				MD		58	4.22 ± 1.04
**Highest educational/vocational qualification**	0.642	0.040	**0.001**	noMD		173	2.4 ± 1.69
				MD		58	3.28 ± 1.67
**Health insurance type** [public/private]	0.546	0.024	**0.023**	NoMD	public	173	95.6
				MD	public	51	86.4
				NoMD	privat	8	4.4
				MD	privat	8	13.6
**Employment contract** [permanent/fixed-term] [b]	0.547	0.011	**0.002**	NoMD	permanent	164	90.6
				MD	permanent	59	100.0
				NoMD	fixed-term	17	9.4
				MD	fixed-term	/	/
**Study center** (place of intervention delivery)	0.689	0.035	**<.001**	NoMD	Berlin	25	13.8
				MD	Berlin	3	3.4
				NoMD	Hamburg	44	24.3
				MD	Hamburg	27	45.8
				NoMD	Rinteln	46	25.4
				MD	Rinteln	3	5.1
				NoMD	Bad B.	17	9.4
				MD	Bad B.	9	15.4
				NoMD	Hanover	49	27.1
				MD	Hanover	18	30.5

NoMD No minimum RTW-PIA dose achived; *MD* minimum dose achieved

[a] Higher values = negative/undesirable; [b] higher values = positive/desirable

[b] Estimates for employment contract should be interpreted with caution due to quasi-complete separation, as there were no fixed-term contracts in this group.

Table S14 Baseline variables significantly predicting whether a participant is in the specific per-protocol intervention group (*n* = 59) vs. control group (*n* = 244)

**Significant Baseline variables**
Cohabitation
Socio-economic status index
Study center
General social support
Highest school-leaving qualification
Highest educational/vocational qualification
Health insurance type
Employment contract
Migrant background

Table S15 Baseline variables significantly predicting whether a participant with complete data across all time points (*n* = 116) is in the intervention group vs. control group

**Significant baseline variables**
Cohabitation
Highest educational/vocational qualification

Table S16 Baseline variables significantly predicting whether a participant with depressive disorder (*n* = 150) is in the intervention group vs. control group

**Significant baseline variables**
Health-related quality of life
Work ability
Depressive symptoms
General social support
Health insurance type

Table S17 Baseline variables significantly predicting whether a participant with recurrent depressive disorder (*n* = 263) is in the intervention group vs. control group

**Significant baseline variables**
Social support (supervisors)
Migrant background
